# SMC4 Promotes Prostate Cancer Cell Proliferation and Metastasis via the Rheb/mTOR Pathway

**DOI:** 10.1002/advs.202500369

**Published:** 2025-04-25

**Authors:** Wei Zhang, Siyuan Qin, Xiaokang Li, Ming Chang, Tianzi Wei, Xin Huang, Haibo Tong, Xia Guo, Yi Lu, Jian Zhang

**Affiliations:** ^1^ School of Medicine Southern University of Science and Technology Shenzhen Guangdong 518053 China; ^2^ Shenzhen Key Laboratory of Cardiovascular Health and Precision Medicine School of Public Health and Emergency Management Southern University of Science and Technology Shenzhen Guangdong 518055 China; ^3^ State Key Laboratory of Biotherapy and Cancer Center West China Hospital and West China School of Basic Medical Sciences & Forensic Medicine and Collaborative Innovation Center for Biotherapy Sichuan University Chengdu Sichuan 610041 China; ^4^ Reproductive Medicine Center Wuhan Jinxin Integrated Chinese and Western Medicine Obstetrics and Gynecology Hospital Wuhan 430000 China; ^5^ Xiangyang Central Hospital Affiliated Hospital of Hubei University of Arts and Science Xiangyang Hubei 441000 China; ^6^ Department of Pulmonary and Critical Care Medicine Shenzhen Institute of Respiratory Diseases First Affiliated Hospital of Southern University of Science and Technology Second Clinical Medical College of Jinan University Shenzhen 518020 China; ^7^ Center for Clinical Research and Innovation (CCRI) Shenzhen Hospital Southern Medical University Shenzhen Guangdong 518101 China; ^8^ Department of Human Cell Biology and Genetics Joint Laboratory of Guangdong‐Hong Kong Universities for Vascular Homeostasis and Diseases School of Medicine Southern University of Science and Technology Shenzhen Guangdong 518101 China; ^9^ SUSTech‐KCL School of Medicine Southern University of Science and Technology Shenzhen Guangdong 518055 China

**Keywords:** GLUT1, metastasis, prostate cancer, SMC4

## Abstract

Structural maintenance of chromosome protein 4 (SMC4), is a key structural component of mitotic chromosomes. While existing evidence indicates a plausible link between SMC4 and oncogenic manifestations, its precise role in the trajectory of prostate cancer remains ambiguous. The Cancer Genome Atlas (TCGA) database analysis reveals that aberrant expression of SMC4 exhibits a robust prognostic association with metastatic progression. To investigate the function of SMC4, the *SMC4* gene is knocked down in RM1‐LM cells, a highly metastatic cell clone is developed, using the CRISPR/Cas9 system. The results show that SMC4 knockdown significantly diminished cell proliferation and migration in vitro. Furthermore, in a murine model, RM1‐LM cells display higher lung metastasis capabilities than SMC4 knockdown cells. SMC4 knockdown inhibited the activation of mTOR and downregulated the expression of Rheb. KEGG enrichment analyses of the RNA‐seq results reveal that cancer signaling pathways and metabolic pathways are enriched. The SMC4 interactome is uncovered through IP‐MS and indicates that SMC4 interacts with GLUT1, encoded by *Slc2a1*. Glycolytic rate assay illustrates that knocking down SMC4 inhibits the cell glycolysis rate and ATP production. Collectively, the data suggests that the interaction between SMC4 and GLUT1, as confirmed by co‐IP, promotes prostate cancer cell metastasis through the Rheb/mTOR pathway.

## Graphical abstract

Structural maintenance of chromosome protein 4 (SMC4) has been implicated in prostate cancer metastasis. Knockdown of SMC4 in RM1‐LM cells significantly attenuates proliferation, migration, and lung metastasis in vivo. Furthermore, SMC4 interacts with GLUT1 (Slc2a1), thereby enhancing glycolysis, ATP production, and activation of the Rheb/mTOR pathway. Elevated levels of SMC4 may potentially serve as a prognostic biomarker in prostate cancer.

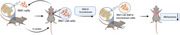

## 1 Introduction

Prostate cancer—with an estimated 288 300 new cases in the United States alone during 2023—is the most frequently diagnosed malignant male tumor, and represents 29% of all new cancer cases in men in the United States.^[^ ^1^ ^]^ Androgen‐deprivation therapy (ADT) is the standard of care for men with prostate cancer, and patients with localized prostate cancer exhibit longer overall survival.^[^ ^2^, ^3^ ^]^ Unfortunately, advanced prostate cancer, known as castration‐resistant prostate cancer (CRPC), remains largely incurable. The lethality of prostate cancer is primarily driven by metastasis.^[^ ^4^, ^5^, ^6^ ^]^ Metastatic prostate cancer continues to be an incurable disease, with 34 700 estimated deaths from prostate cancer in the United States in 2023, which represents 11% of all cancer deaths in men.^[^ ^1^ ^]^ The most common site of prostate cancer metastasis is bone, with up to 84% of patients demonstrating skeletal metastases.^[^ ^7^ ^]^ Despite recent progress, prostate cancer remains a significant medical problem for the men affected, with frequent overtreatment of inherently benign disease and inadequate therapies for metastatic prostate cancer, and with the mechanism(s) by which prostate cancer metastasizes still being unclear.

Structural maintenance of chromosomes protein 4 (SMC4) is a member of the SMC family, which is comprised of chromosomal ATPases.^[^ ^8^ ^]^ These ATPases are highly conserved from bacteria to humans, maintain the stability of chromosomal structure, and participate in the mitoses of eukaryotic cells.^[^ ^9^ ^]^ The principal function of SMC4 lies in assisting in the process of chromosomal transition from loose interphase chromatin to the agglomerated state, and in sister‐chromatid separation during cell division. In addition, SMC4 also plays a significant role in the non‐dividing phase of the cell cycle, including the maintenance of gene repression, heterochromatin organization, and DNA repair. Previous studies showed that the expression level of SMC4 was abnormally high in the pathogenesis and progression of lung cancer, liver cancer, breast cancer, and colon cancer.^[^ ^10^, ^11^, ^12^, ^13^ ^]^ SMC4 overexpression also promoted lung adenocarcinoma progression and acted as an independent prognostic factor,^[^ ^10^ ^]^ and SMC4‐mediated glioma cell aggressiveness ensued via the TGFβ/Smad‐signaling pathway.^[^ ^14^ ^]^ In breast cancer cells, SMC4 regulates expression of the P53 pathway genes, and this may relate to the alterations in chromosomal stability. Although elevated expression of SMC4 is significantly associated with the metastatic cascade and offers a poor prognosis in prostate cancer,^[^ ^15^ ^]^ its expression and potential role in the pathogenesis of prostate cancer remain obscure.

In our study, the expression of SMC4 was increased in a prostate cancer cell line with high lung metastatic specificity (RM1‐LM), an organ‐specific metastatic cell line that we developed. To investigate the function of SMC4, we knocked down the *SMC4* gene in RM1‐LM cells with CRISPR/Cas9; and also analyzed the expression of SMC4 in clinical tissues and found that it related to Gleason grade. Blocking SMC4 suppressed cell proliferation and migration in vitro and reduced cell metastasis and progression in mice. Furthermore, we explored the mechanism by which SMC4 promotes prostate cancer cell metastasis.

## 2 Results

### 2.1 High SMC4 Expression Is Associated with Poor Prognosis and the Gleason Grade in Human Prostate Cancer

**Figure 1:**
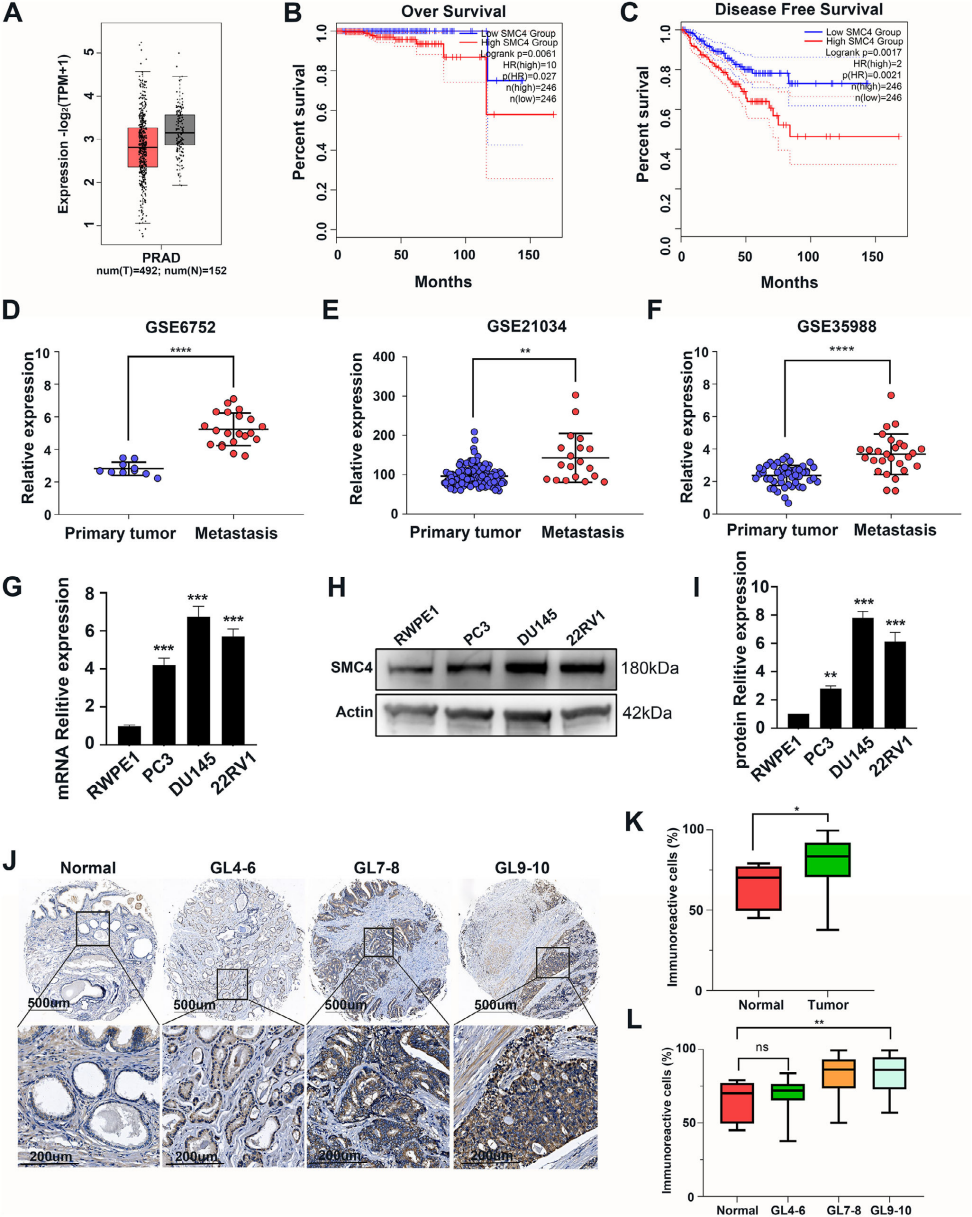
SMC4 expression is upregulated in human prostate cancer and related to the poor prognosis and metastasis. A) Comparison of SMC4 gene expression between prostate cancer tissues and normal tissues in a TCGA dataset. B) Kaplan‐Meier curves of overall survival in TCGA prostate cancer patients with SMC4 high or low expression divided by the median. Data are from the Oncomine database. C) Kaplan‐Meier curves of disease‐free survival in TCGA prostate cancer patients with SMC4 high or low expression divided by the median. Data are from the Oncomine database. D—F) SMC4 mRNA is upregulated in metastases relative to primary tumors in Oncomine prostate cancer datasets (**p < 0.01, ****p < 0.0001 based on the Student's *t* test; data are presented as means ± SD). G) The mRNA expression in human prostate epithelial and prostate cancer cell lines. H) Western blot showing the expression of SMC4 in human prostate epithelial and prostate cancer cell lines. I) Relative expression of SMC4 mRNA in human prostate epithelial and prostate cancer cell lines. J) IHC staining of SMC4 protein within a human prostate cancer tissue microarray (scale bar represents 200 µm). K) Statistical comparison of SMC4 protein expression between normal and tumor patients. L) Statistical comparison of SMC4 protein expression based on Gleason scores (*p < 0.05, **p < 0.01 based on Student *t* test; data represent means ± SD).

To explore the clinical role of SMC4, we analyzed its expression in various types of tumors using data from The Cancer Genome Atlas (TCGA) database. The findings showed that SMC4 is highly expressed in multiple cancers (Figure S1, Supporting Information). We further investigated 492 prostate cancer tissues and 152 normal prostate tissues, and the results demonstrated that there was no difference in the expression of SMC4. (**Figure** **1**A). However, survival analyses revealed that overall survival was significantly lower in the high‐SMC4 group compared to the low‐SMC4 group (*p* < 0.01) (Figure 1B). Moreover, patients with higher SMC4 mRNA expression manifested poor disease‐free survival compared with those with lower SMC4 mRNA expression, and results were identical for overall survival (*p* < 0.01) (Figure 1C). Furthermore, our examination of the Oncomine prostate cancer datasets revealed a noteworthy elevation of SMC4 mRNA expression in metastatic prostate cancer compared to that in primary tumors, suggesting an ongoing contribution to the progression of metastasis (Figure 1D–F).

We measured the expression of SMC4 in human prostate cancer cell lines. The qPCR and western blotting results showed that SMC4 was significantly overexpressed in these cell lines (Figure 1G–I). IHC results from human prostate cancer tissue microarrays indicated that SMC4 protein expression was elevated in prostate cancer tissues in tandem with increased Gleason grade (Figure 1J–L). Our findings indicate that outlier expression of SMC4 exhibit a robust prognostic association with metastatic progression, and that the SMC4 prognostic outlier genes continue to occupy roles in metastatic prostate cancer.

### 2.2 SMC4 Promotes Human Prostate Cancer Cell Proliferation, Migration, and Invasion in Vitro

**Figure 2:**
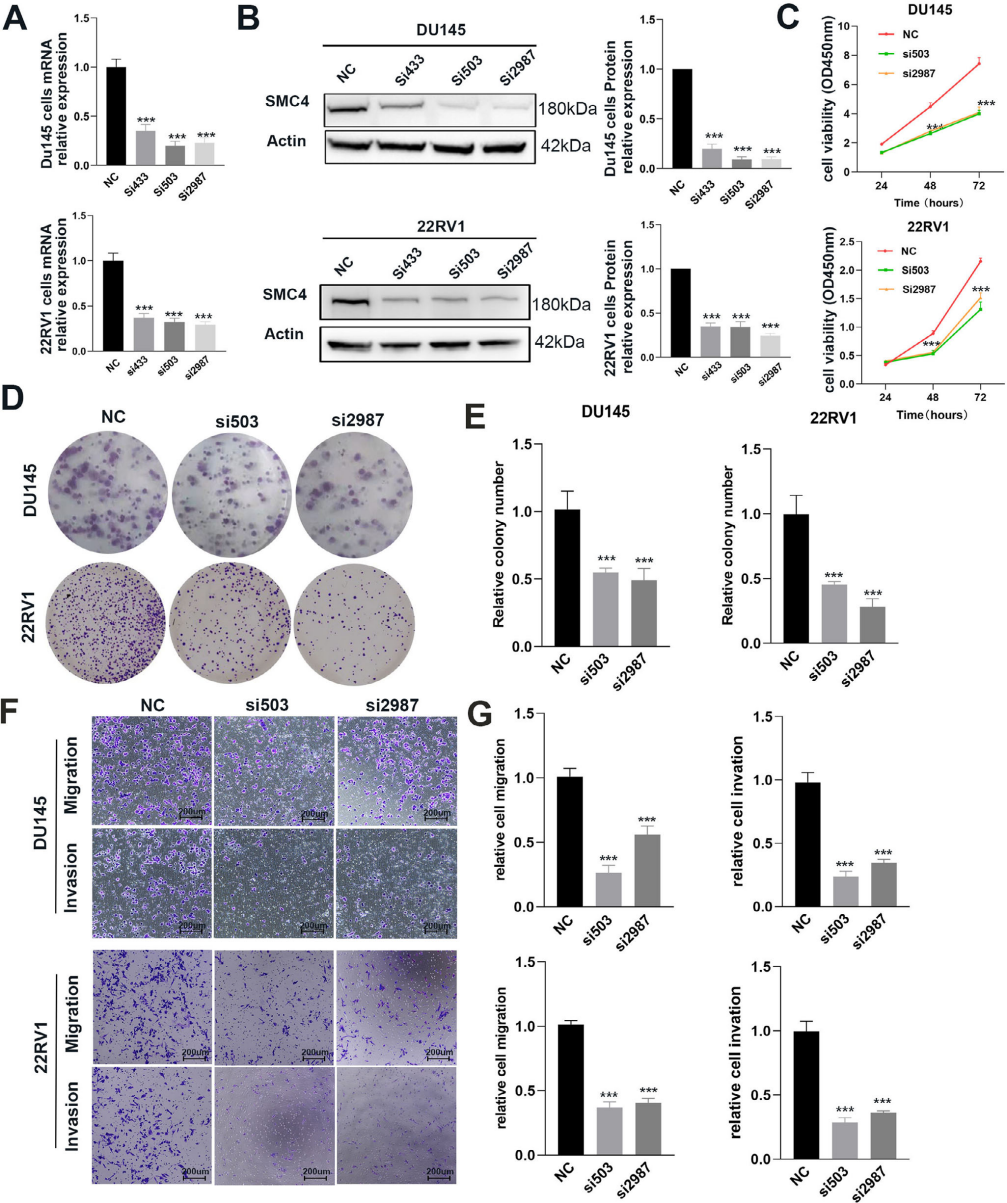
SMC4 promotes human prostate cancer cell proliferation, migration, and invasion in vitro. A,B) siRNA‐mediated knockdown of SMC4 in DU145 and 22RV1 cell lines was verified by real‐time PCR and western blotting. C) A CCK8 assay was executed to measure the proliferation of DU145 and 22RV1 cells after SMC4 knockdown. D) A colony‐formation assay showed that the cells’ proliferative capacity was inhibited after SMC4 knockdown. E) Quantitative analysis of colony numbers. F) Migration and invasion after siRNA‐mediated knockdown of SMC4 in DU145 and 22RV1 cells. G) Relative analysis of migration and invasion. ***p < 0.001 based on Student *t* test; error bars signify means ± SEM of three independent experiments.

To elucidate the functional roles of SMC4, we employed siRNA‐SMC4 transfection to suppress SMC4 expression in the DU145 and 22RV1 cell lines. Quantitative PCR (qPCR) and Western blotting (WB) confirmed the successful knockdown of SMC4 (**Figure** **2**A,B). Subsequently, we assessed cell proliferation capacity and colony formation ability post‐transfection and observed a significant inhibition of growth upon SMC4 knockdown (Figure 2C–E). In addition, we performed a Transwell assay to evaluate cellular migratory and invasive capabilities following transfection (Figure 2F). Notably, DU145 and 22RV1 cells transfected with siRNA displayed lower migration and invasion capabilities than the controls (*P* < 0.01, Figure 2G).

### 2.3 SMC4 Promotes Mouse Metastatic Prostate Cancer Cell Proliferation, Migration, and Invasion Capabilities in Vitro

**Figure 3:**
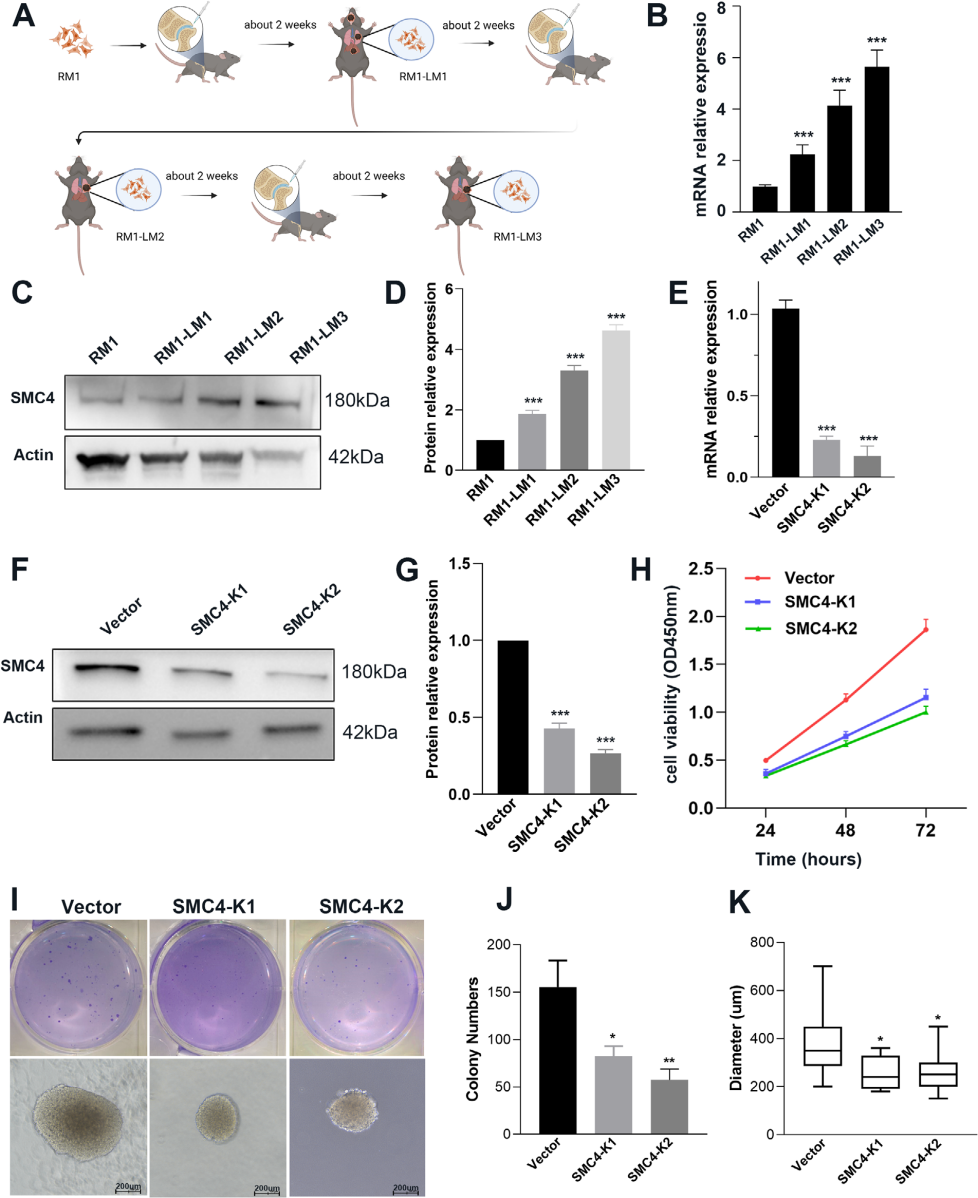
SMC4 knockdown via the CRISPR/Cas9 system inhibits highly metastatic prostate cancer cellular proliferation and colony formation. A) Diagram illustrating the isolation of highly metastatic prostate cancer cells. B) The mRNA level of SMC4 was measured by quantitative real‐time PCR in RM1cells and highly metastatic prostate cancer cells. C) Western blotting results showing SMC4 expression in RM1 cells and highly metastatic prostate cancer cells. D) The relative protein expression of SMC4 in RM1 cells and highly metastatic prostate cancer cells. E) Knockdown of SMC4 was confirmed by quantitative real‐time PCR. F) Knockdown of SMC4 was confirmed by western blotting. G) The relative protein expression of SMC4 in RM1‐LM‐vector and SMC4 knockdown cells. H) CCK8 assay was executed to measure proliferation of RM1‐LM‐Vector and SMC4 knockdown cells. I) We used colony‐formation assays to evaluate proliferative capacity in RM1‐LM‐Vector and SMC4 knockdown cells. J) Quantitative analysis of colony numbers. K) Quantitative analysis of diameter.*p < 0.05, **p < 0.01, ***p < 0.001. Data are shown as the mean ± SD.

To determine the functional effects of SMC4 on metastatic prostate cancer cells, we inoculated RM1 cells into the tibiae of C57BL/6J mice and then reinjected lung metastatic tumor cells into shin bones of the mice. After three cycles of repetition, we obtained a stable population of highly metastatic lung cells (RM1‐LM) (**Figure** **3**A). The expression of SMC4 in RM1‐LM cells versus RM1 cells was assessed by qPCR and western blotting. SMC4 expression was found to be significantly higher in RM1‐LM cells than in RM1 cells (Figure 3B–D).

**Figure 4:**
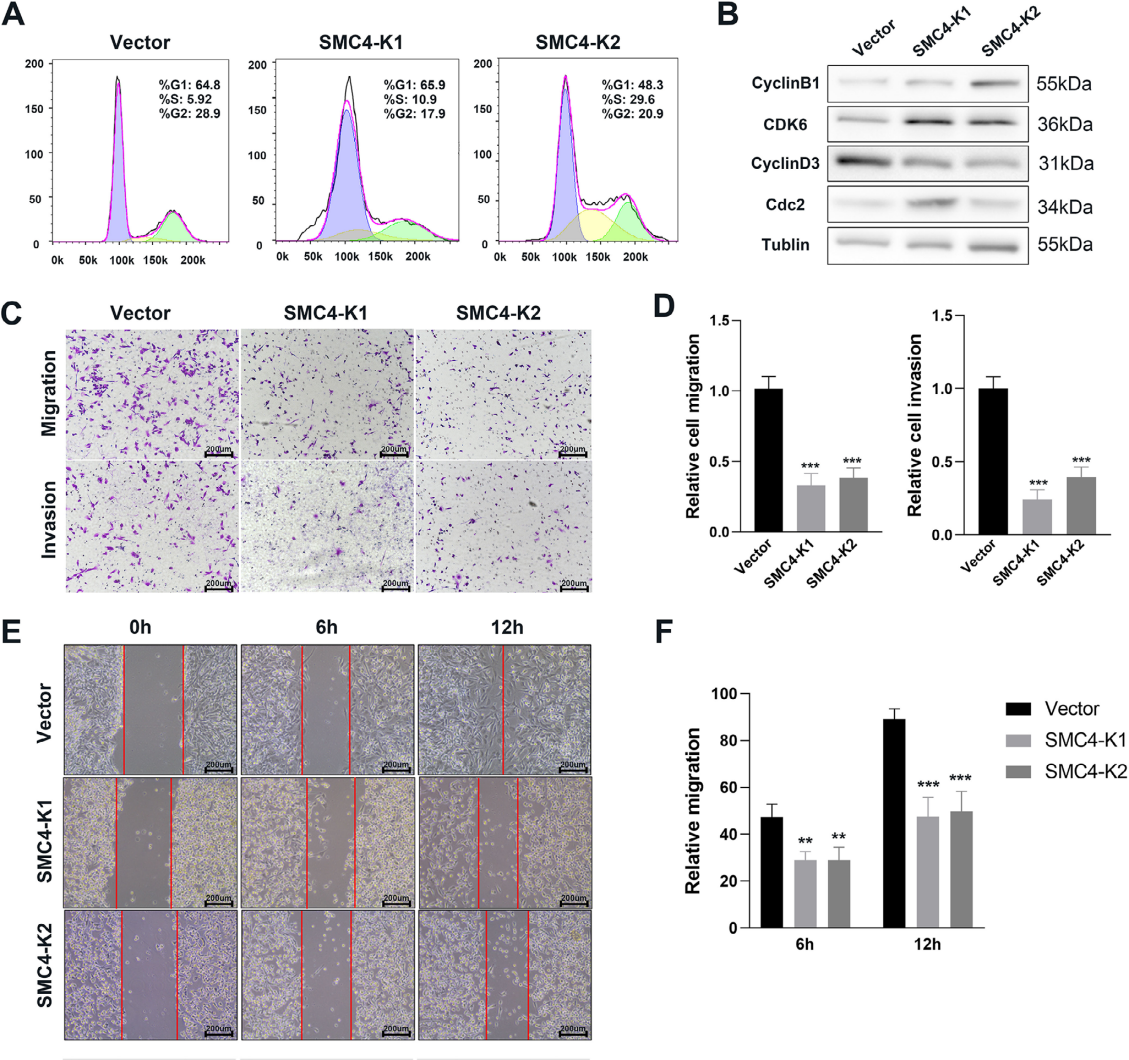
SMC4 knockdown inhibits cell cycle progression and cell migration and invasion, promoting mouse prostate cancer metastasis and progression in vitro. A,B) Flow cytometry and western blot analysis were used to assess cell cycle kinetics. C) Transwell assays were performed to determine cell migration and invasion. D) Relative analysis of migration and invasion. E) Images of wound‐healing assay. F) The area of cell migration into the scratch wound compared with that at 0 h, which was determined as 100% open. **p < 0.01, ***p < 0.001. Data are shown as the mean ± SD.

We performed SMC4 knockdown using the CRISPR/Cas9 system in RM1‐LM cells. The identification of SMC4‐knockdown colonies (SMC4‐K1 and K2) was performed via qPCR and western blotting (Figure 3E–G). Subsequently, various phenotypic features were compared between SMC4 knockdown cells and transfected empty vector control cells. We evaluated cell growth and colony formation in vector cells alongside SMC4 knockdown cells. CCK8 assay and colony formation experiments indicated a significant reduction in the proliferation of cells upon SMC4 knockdown (Figure 3H–J). Furthermore, the diameter of the cell colonies was significantly smaller after SMC4 knockdown (Figure 3K). In addition, the effect of SMC4 on cell DNA synthesis and cell cycle progression was investigated through flow cytometry analysis. SMC4 knockdown significantly decreased the number of cells in the G2/M phase and increased the number in the S phase (**Figure** **4**A). The expression of cyclin‐dependent kinases and cyclin proteins was also measured using western blot (Figure 4B). The Transwell assay demonstrated a remarkable decrease in cellular migration and invasion upon SMC4 knockdown (Figure 4C,D). Consistently, the wound healing assay indicated a prominently suppressed cell migration rate after SMC4 knockdown (Figure 4E,F). In contrast, cell proliferation, migration, and invasion capabilities were increased after SMC4 overexpression in knockdown cells (Figure S2, Supporting Information). Thus, SMC4 knockdown can effectively suppress metastatic prostate cancer cells’ growth, migratory, and invasion capabilities.

### 2.4 SMC4 Promotes Prostate Cancer Metastasis and Progression in Vivo

**Figure 5:**
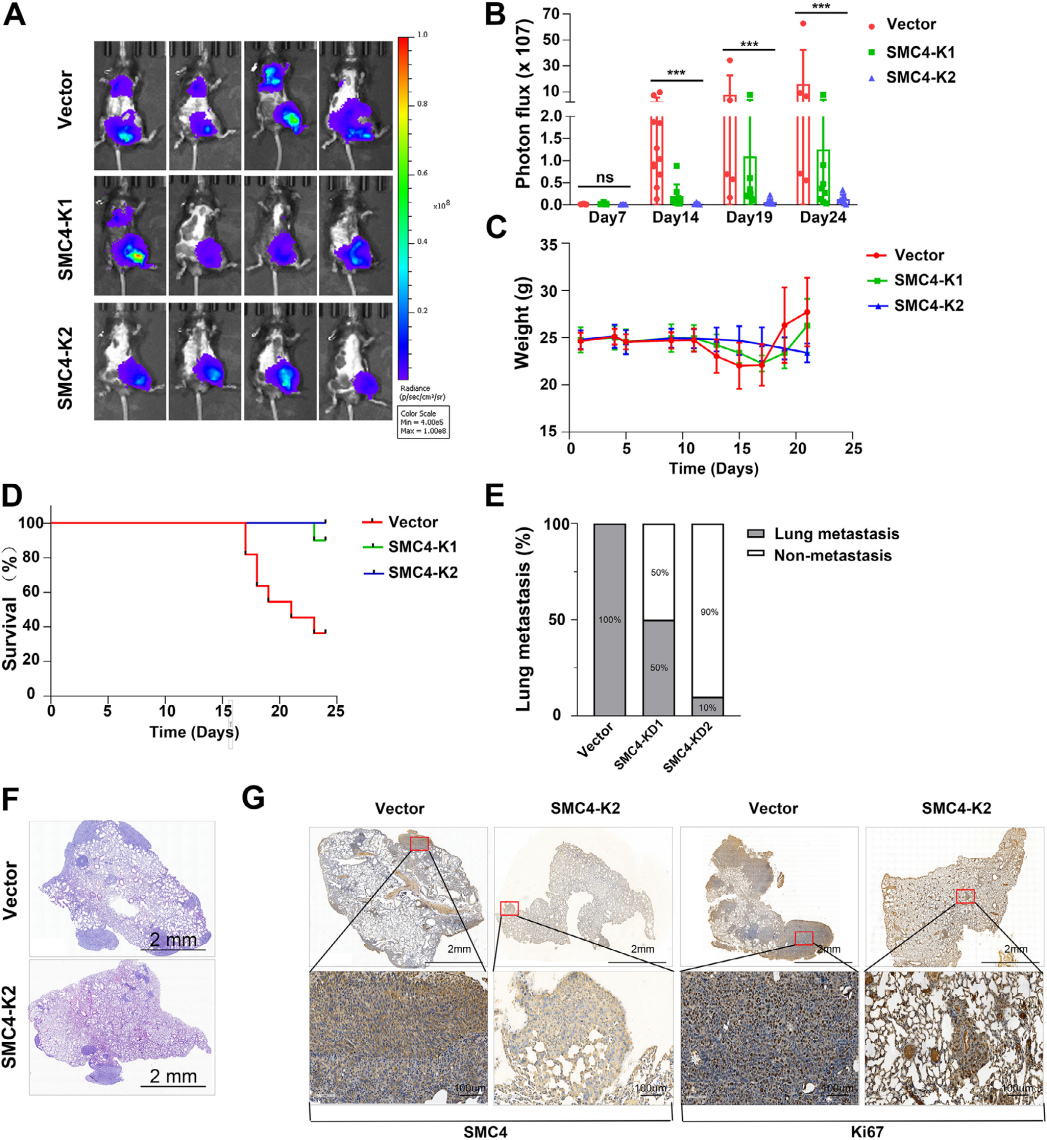
SMC4 knockdown inhibits highly metastatic prostate cancer cell metastasis and progression in vivo. A) Bioluminescence imaging (BLI) of mouse tumors was measured every 5–7 days, and the images are from day 24th. N (vector) = 11, N (SMC4‐K1/K2) = 10. B) Quantification of tumor photon flux in lung metastases in mice. C) Mouse weights were measured every 2–3 days. D) Kaplan‐Meier curves show mouse survival after inoculation with RM1‐LM‐Vector cells or SMC4‐knockdown cells. E: Percent lung metastasis of RM1‐LM‐Vector cells and SMC4‐knockdown cells after inoculation. F) Mouse lung tissues were harvested, fixed, embedded in paraffin, and stained with H&E. G) SMC4 and Ki‐67 expressions were detected by IHC staining of lung tissues. ***p < 0.001. Data are shown as the mean ± SD.

To investigate the contribution of SMC4 to prostate cancer growth and metastasis, we inoculated RM1‐LM‐Vector cells and SMC4 knockdown cells into the tibia of C57BL/6 mice, and 14 days after injection, the mice inoculated with RM1‐LM‐Vector cells showed dramatic increases in bioluminescent imaging (BLI) signals compared with mice inoculated with SMC4 knockdown cells (**Figure** **5**A). We normalized the lung BLI signal to the higher BLI signal in mice inoculated with RM1‐LM cells compared to mice inoculated with SMC4 knockdown cells (Figure 5B), and our data revealed that the majority of BLI signals in the lung corroborated pulmonary metastasis. In contrast, mice inoculated with SMC4 knockdown cells showed lower BLI signals (Figures S3–S5, Supporting Information). Mouse weights were measured every 2–3 days (Figure 5C), and between days 17 and 23, seven mice inoculated with RM1‐LM‐Vector cells died; in contradistinction, only one mouse inoculated with SMC4 knockdown cells died (Figure 5D). Compared to the SMC4 knockdown cells, the RM1‐LM‐Vector cells showed enhanced lung metastasis (Figure 5A,E), and our H&E and IHC results revealed that RM1‐LM‐Vector cells increased lung metastasis and colonization relative to SMC4 knockdown cells, suggesting that loss of SMC4 suppressed prostate cancer metastasis in addition to tumor growth (Figure 5F,G).

### 2.5 Transcriptomic Profiling of RM1‐LM and SMC4 Knockdown Cells

**Figure 6:**
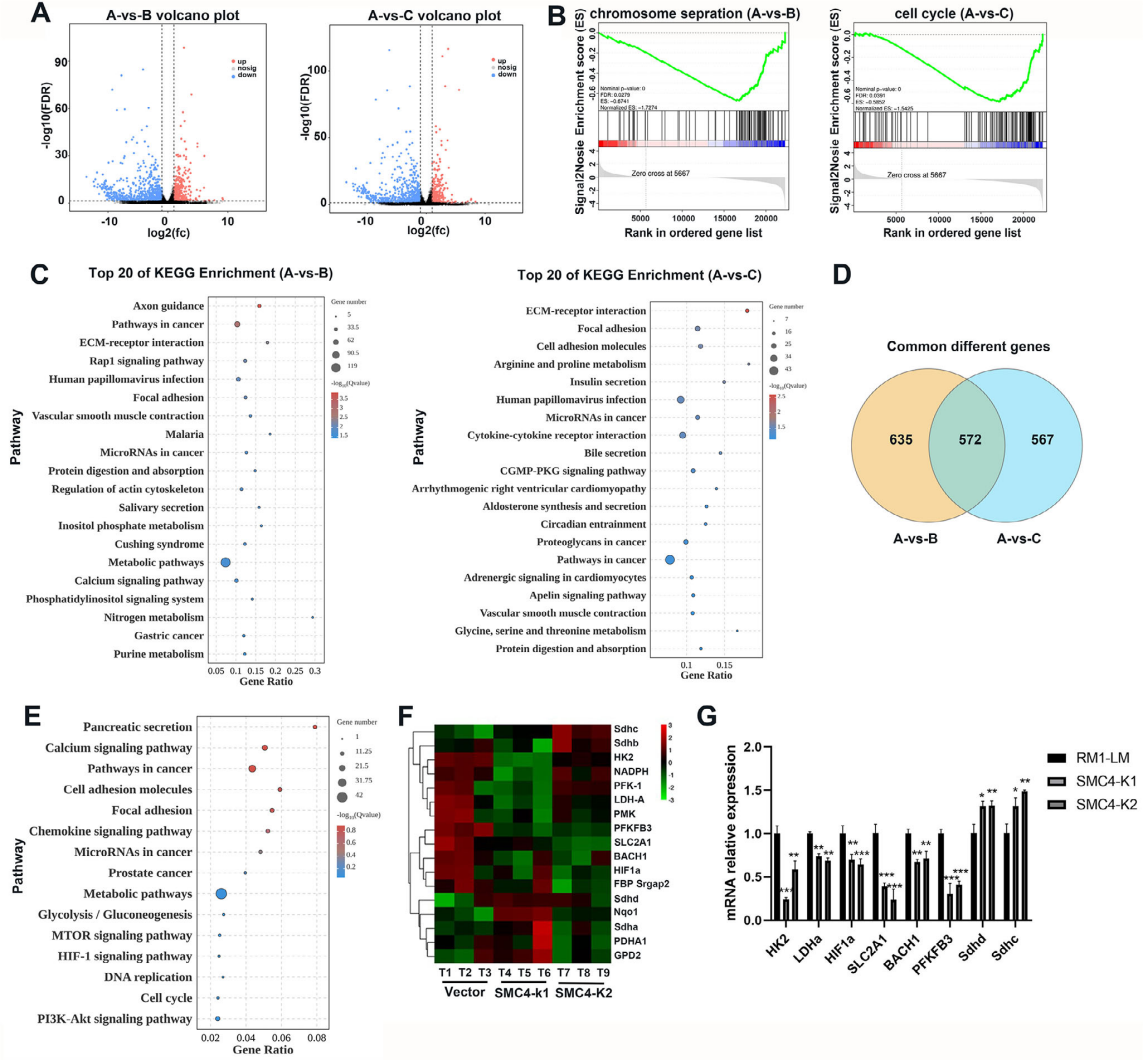
Transcriptomic profiling of RM1‐LM and SMC4 knockdown cells. A) Volcano plots show the differential expression of mRNA transcripts between RM1‐LM‐Vector and SMC4‐knockdown cells. Red, gray, and blue represent significantly upregulated, non‐significant, and significantly downregulated mRNAs, respectively. A: RM1‐LM‐Vector cells. B) SMC4‐K1 cells. C: SMC4‐K2 cells. B) GSEA shows significant enrichment of gene‐expression signatures in the cell cycle and chromosomal separation. FDR, false‐discovery rate; normalized ES, normalized enrichment score. C) The top genes in the Kyoto Encyclopedia of Genes and Genomes (KEGG) signaling‐pathway enrichment in differentially expressed gene (DEG) sets. The color of the scattered dots varies from blue to red and designates a range of significant differences, and circle size indicates the number of enriched DEGs. D) The common DEGs enriched in RM1‐LM‐Vector versus SMC4‐K1 and RM1‐LM‐Vector versus SMC4‐K2 cells. E) The common DEGs identified by KEGG analysis. F) Heatmap of the mRNA expression of glycolytic genes in the RNA‐Seq analysis in RM1‐LM3 and SMC4‐knockdown cells. Red and green denote upregulated and downregulated mRNA expression, respectively. We conducted cluster analysis for sample and differential mRNAs. G) The qPCR results in RM1‐LM and SMC4 knockdown cells. *p < 0.05, **p < 0.01, ***p < 0.001. Data are shown as the mean ± SD.

To elucidate the mechanism responsible for the SMC4 promotion of prostate cancer cell metastasis, we performed RNA‐Seq analysis in RM1‐LM‐Vector and SMC4 knockdown cells. Our volcano map depicts differential gene expression between RM1‐LM‐Vector and SMC4 knockdown cells (**Figure** **6**A). There were 396 genes upregulated and 811 genes downregulated in RM1‐LM‐Vector cells versus SMC4‐K1 cells, and 349 genes upregulated and 790 genes downregulated in RM1‐LM‐Vector cells versus SMC4‐K2 (Figure S6, Supporting Information). To further determine the signaling pathways most impacted by SMC4, we exploited gene set enrichment analysis (GSEA) on the differentially expressed genes (DEGs). SMC4 is a key component of condensin and plays a crucial role in chromosomal transition and sister‐chromatid separation during cell division. As shown in Figure 6B, DNA replication and cell cycle progression‐related genes were enriched for downregulation in SMC4 knockdown cells as expected. Also, Kyoto Encyclopedia of Genes and Genomes (KEGG) enrichment analysis revealed numerous pathways activated after SMC4 knockdown. The top 20 gene sets that were enriched included pathways in cancer, focal adhesion, metabolic pathways, and insulin secretin (Figure 6C). We further integrated the RNA‐Seq data to obtain common DEGs between RM1‐LM‐Vector versus SMC4‐K1, and RM1‐LM‐Vector versus SMC4‐K2. A total of 572 common DEGs were enriched (Figure 6D). The KEGG‐enrichment results showed that genes related to cell and glucose metabolism were significantly enriched in common DEGs (Figure 6E). Furthermore, the ablation of SMC4 altered the mRNA expression of cellular glucose metabolism‐related genes (Figure 6F,G). The depletion of SMC4 greatly changed the cellular metabolic state.

### 2.6 SMC4 Interacts with and Modulates the Glycolytic Metabolic Profile in PCa (RM1) Cells

**Figure 7:**
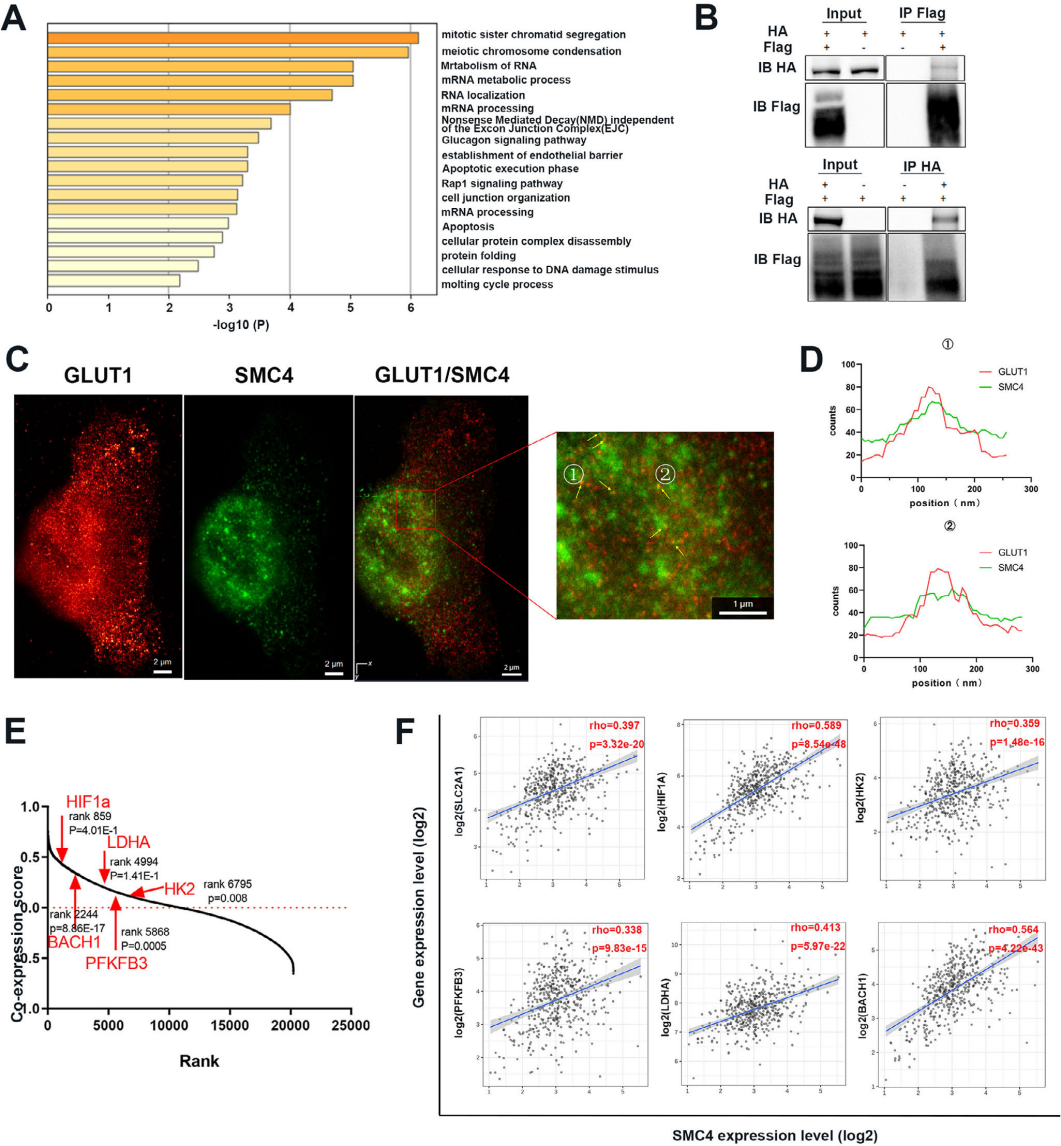
SMC4 interacts with GLUT1 in RM1 cells. A) SMC4‐interacting proteins by LC‐MS/MS analysis were enriched according to Kappa‐statistical similarities. B) SMC4 was labeled with HA, and GLUT1 was labeled with Flag; co‐IP showed that SMC4 interacted with GLUT1. C) Immunofluorescence staining. Scale bars, 2 µm. The areas indicated by boxes are magnified below. D) Comparison of fluorescence intensity profiles. E) Co‐expression of SMC4, BACH1, HK2, PFKFB3, and SLC2A1 in human prostate cancer. Data are from the TCGA dataset. F) The correlation between SMC4 expression and GLUT1, HK2, HIF1a, LDHA, and BACH1 was analyzed using the TCGA dataset.

We performed IP assays using anti‐flag magnetic beads and identified SMC4‐interacting proteins by LC‐MS/MS analysis. A total of 76 proteins were identified as interacting specifically with Flag‐SMC4 (Table S1, Supporting Information). We hierarchically clustered the remaining significant terms according to Kappa‐statistical similarities among their gene memberships (**Figure** **7**A). Notably, *SLC2A1*, which encodes the major glucose transporter 1 (GLUT1) The Cancer Genome Atlas (TCGA) and plays a crucial role in the glycolytic pathway, was enriched in the Glucagon signaling pathway. Since the RNA‐Seq results indicated an effect on cellular metabolic signaling pathways after SMC4 knockdown, we selected GLUT1 for further validation by co‐IP (Figure 7B). We performed immunofluorescence staining to evaluate the interaction (Figure 7C,D) more directly. Consistent with the co‐IP results, we found that SMC4 and GLUT1 are associated.

SMC4 knockdown suppressed transcript levels of glycolytic genes that included 6‐phosphofructo‐2‐kinase/fructose‐2,6‐bisphosphatase 3 (*PFKFB3*), solute‐carrier family 2 member1 (*Slc2a1*), hypoxia‐inducible factor 1 subunit alpha (*HIF1a*), hexokinase 2 (*HK2*), lactate dehydrogenase A (*LDHA*), and BTB domain and CNC homolog1 (*BACH1*) (Figure 6F,G). Furthermore, co‐expression analyses in TCGA datasets characterized SMC4 expression as correlated with glycolytic genes (Figure 7E). Subsequently, utilizing the TCGA database on the TIMER website, we analyzed the association between SMC4 and these genes. The findings illustrated a significant positive correlation between SMC4 and SLC2A1, HIF1a, HK2, PFKFB3, LDHA, and BACH1 (Figure 7F).

### 2.7 SMC4 Knockdown Affected the Glycolysis of Metastatic Mouse Prostate Cancer Cells

**Figure 8:**
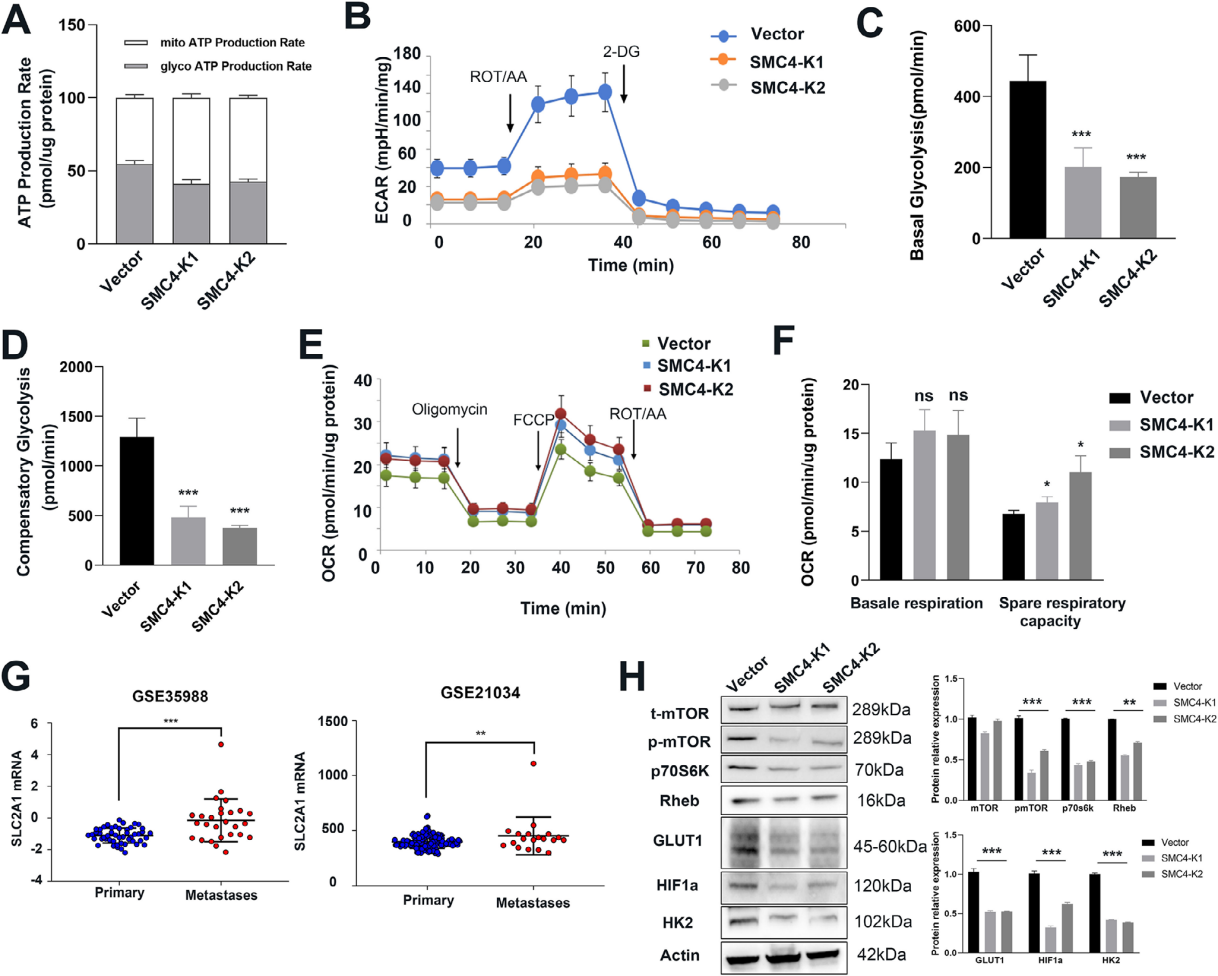
The effect of SMC4 on glycolysis rates and mitochondrial respiration. A) ATP production rate assay of RM1‐LM‐Vector and SMC4‐knockdown cells. B—D) SMC4 knockdown inhibits RM1‐LM‐Vector cell glycolytic rates. The basal and compensatory glycolysis rates were significantly lower after SMC4 knockdown. E,F) Mito stress test results demonstrated that cells’ spare respiratory capacity was higher after SMC4 knockdown. G: *SLC2A1* was upregulated in metastases versus primary tumors in the Oncomine prostate cancer datasets. H) Western blotting results of mTOR pathway and glycolysis proteins in cells. *p < 0.05, **p < 0.01, ***p < 0.001. Data are shown as the mean ± SD.

Next, we evaluated the association between SMC4 and glycolysis using a Seahorse XFe96 analyzer. The ATP rate assay was performed and exhibited a reduction in ATP production from glycolysis upon SMC4 knockdown (**Figure** **8**A). Conversely, ATP production through mitochondrial respiration was increased in SMC4 knockdown cells. Moreover, the glycolysis rates assay indicated a significant decline in basal and compensatory glycolysis within SMC4 knockdown cells versus RM1‐LM‐Vector cells (Figure 8B–D). The mito stress test revealed elevated spare respiratory capacity after SMC4 knockdown (Figure 8E,F). In addition, analyses of GSE21034 and GSE35988 databases revealed that GLUT1 expression was augmented in metastatic prostate cancer in men relative to that in primary prostate cancer, a trend consistent with SMC4 expression (Figure 8G). These metabolite alterations, including ATP, suggested that SMC4 knockdown suppressed glycolysis rates.

Reports show that the mTOR pathway is important in glycolysis.^[^ ^16^, ^17^ ^]^ Because of the association between SMC4 and GLUT1, we sought to explore whether SMC4 regulates glycolysis by activating the mTOR pathway in RM1 cells. We examined the proteins involved in mTOR pathways. Western blot analysis revealed a decrease in phosphorylation of mTOR following SMC4 knockdown, while the total mTOR protein quantity remained unchanged (Figure 8H). Moreover, the upstream protein Rheb exhibited a significant decrease in expression, indicating that SMC4 may modulate cell proliferation and migration by regulating the Rheb/mTOR pathway. Meanwhile, we found that the expression of genes involved in glycolysis, including GLUT1, HK2, and HIF1a, was partly decreased. Cell migration and invasion capacities were increased when SMC4‐K1 and K2 cells were treated with an mTOR activator MHY1485 (Figure S7, Supporting Information). Thus, SMC4 potentially regulates the growth and metastasis of metastatic prostate cancer cells through the Rheb/mTOR pathway.

## 3 Discussion

Despite the high long‐term survival rate in localized prostate cancer, advanced tumors are incurable even after intensive multimodal therapy, and metastatic disease is the leading cause of death in prostate cancer. Lymph nodes are usually the first sites of metastases for prostate tumors, followed by metastases to the lungs, liver, and bones.^[^ ^18^ ^]^ In this study, we used a prostate cancer cell line that shows prominent metastases to the lungs (RM1‐LM, an organ‐specific metastatic cell line that we developed) to detect underlying tumor mechanisms (Figure 3A), and found that SMC4 was highly expressed in RM1‐LM cells. SMC4 protein expression was also commensurately elevated with increasing injection cycles. This suggested that SMC4 was important in prostate cancer cell metastasis.

The SMC4 protein is a member of the ATPase superfamily.^[^ ^8^ ^]^ As a sensitive switch within the condensin complex, the SMC4 protein interacts with the SMC2 monomer to form a V‐shaped structure.^[^ ^19^ ^]^ Condensin is crucial in cell division processes, facilitating chromosomal condensation, proper compression, and dissociation of sister chromatids.^[^ ^9^, ^20^ ^]^ Knockdown of SMC4 gene expression revealed its essential roles in parasite proliferation and transmission.^[^ ^19^ ^]^ Depletion of SMC4 leads to cytokinesis failure and polyploidy, impeding cell proliferation.^[^ ^21^ ^]^ Previous studies revealed that SMC4 was overexpressed in colorectal, liver, hepatocellular carcinoma, and glioma cells, indicating its significant role in tumor aggressiveness.^[^ ^11^, ^12^, ^13^, ^14^ ^]^ SMC4 was also reported to be involved in a poor prognosis in prostate cancer, lung adenocarcinoma, and acute myeloid leukemia.^[^ ^10^, ^15^, ^22^ ^]^ The Oncomine database depicts SMC4 mRNA levels as much higher in metastases than in primary tumors, indicating a contributing role of SMC4 in metastatic prostate cancer (Figure 1D–F). SMC4 overexpression also promotes cancer cell aggressiveness and metastasis in human prostate cancer cell lines (Figure 2). Such trends have been observed in parallel across diverse cell lineages. Therefore, to determine the functional effect of SMC4 on metastatic prostate cancer cells, we knocked down SMC4 in RM1‐LM cells and demonstrated that SMC4 knockdown notably inhibited cellular proliferation, migration, and invasion in vitro and affected cell cycle. Also, SMC4 knockdown dramatically decreased lung metastasis and colonization relative to RM1‐LM‐Vector cells, suggesting that SMC4 promoted prostate cancer metastasis in addition to tumor growth. Furthermore, when SMC4 is overexpressed, it neutralizes the reduced cell proliferation, migration, and invasion capacities due to SMC4 depletion (Figure S2, Supporting Information).

Nevertheless, the contribution of SMC4 to prostate cancer metastasis remains unelucidated. Jiang et al. verified that SMC4 promoted glioma cell aggressiveness and activated TGF/Smad signaling and that *SMC4* was a target gene regulated by microRNA‐124‐5p in colorectal cancer.^[^ ^12^, ^14^ ^]^ Chen et al. also demonstrated that SMC4 was transcriptionally regulated by hypoxia‐inducible factor‐1 (HIF‐1) under hypoxic conditions in hepatocellular carcinoma.^[^ ^23^ ^]^ In this study, we found that Rheb and p‐mTOR expression levels were increasingly decreased in SMC4‐knockdown cells (Figure 8H). Increasing evidence suggests that mTOR signaling pathways play an important role in cancer cell proliferation, migration, invasion, and glucose metabolism. Thus, SMC4 potentially regulates the growth and metastasis of metastatic prostate cancer cells through the Rheb/mTOR pathway.

We analyzed transcription after SMC4 knockdown and showed that the cell metabolic pathway was enriched based on KEGG functional annotation and GO analysis. Our findings revealed that SMC4 knockdown suppressed the expression of glycolysis‐related genes such as *HK2*, *HIF1A*, *BACH1*, and *PFKF*B3. Studies have demonstrated that targeting glycolysis is one potential strategy to combat metastasis.^[^ ^24^, ^25^, ^26^ ^]^ The Warburg effect was first reported in rat liver carcinoma in the 1920s. Later, researchers found that enhanced aerobic glycolysis in many other cancer types, including prostate, lung, breast, and pancreatic cancer.^[^ ^27^, ^28^, ^29^, ^30^ ^]^ Hexokinase (HK) is the first rate‐limiting enzyme in aerobic glycolysis. Many researchers have investigated how HK2 promotes glycolysis.^[^ ^21^, ^31^ ^]^ DeWaal et al. found that HK2 silencing can synergistically enhance the sensitivity of HCC to sorafenib.^[^ ^31^ ^]^ HK2 attenuation enhances the sensitivity of HCC to drugs such as metformin by inhibiting glycolytic flux and inducing OXPHOS.

Hypoxia‐inducible factor‐1 (HIF‐1) functions as a master regulator of cellular and systemic homeostatic responses to hypoxia and activates the transcription of numerous genes that include those involved in energy metabolism, angiogenesis, apoptosis, and other genes whose protein products increase oxygen delivery or facilitate metabolic adaptation to hypoxia. Previous studies proved that HIF1A was critical to cellular proliferation, migration, and drug resistance in a hypoxic microenvironment.^[^ ^32^, ^33^ ^]^ Notably, Wiel et al. found that BACH1 binds to the HK2 and GAPDH promoters and stimulates the expression of several glycolytic genes, increasing glucose uptake and glycolytic rate. These authors suggested that targeting BACH1 or proteins inhibited lung cancer metastasis.^[^ ^34^ ^]^

Oncogenic mutations have been shown to activate glycolytic enzymes and increase glucose transport into cells. During glycolysis, these oncogenes upregulate GLUT1 expression in cancer cells.^[^ ^35^, ^36^, ^37^ ^]^ In our study, co‐IP results revealed that SMC4 was associated with GLUT1, a major glucose transporter in mammals that is encoded by the SLC2A1 gene (Figure 7B). Furthermore, we correlated the elevated expression of SMC4 with a commensurately augmented expression of GLUT1 in prostate cancer metastasis, and cellular metabolic assays revealed that SMC4 knockdown suppressed glycolytic rate. These data suggested that SMC4 overexpression promoted cancer cell metastasis by modifying cellular metabolism.

In summary, our findings illuminated the profound influence of SMC4 overexpression on the heightened aggressiveness of prostate cancer cells, specifically in facilitating their metastatic dissemination to distant organs. Our investigation uncovered the suppression of mTOR activity and Rheb expression after SMC4 knockdown, suggesting the potential role of SMC4 in governing the growth and metastasis of prostate cancer cells through the Rheb/mTOR signaling pathway. The association between SMC4 and GLUT1 was confirmed by co‐IP, providing new insights into the mechanism underlying metastatic prostate cancer. Targeting glycolysis could potentially develop into a strategy to inhibit cellular proliferation and migration in prostate cancers. Notably, the elevated expression levels of SMC4 demonstrate a noteworthy correlation with overall patient survival, thus rendering it a potentially valuable prognostic marker for prostate cancer.

## 4 Experimental Section

### Cell culture, Plasmid Construction, and Transfection

RM1, DU145, and 22RV1 cell lines were obtained from the American Type Culture Collection (Manassas, VA, USA). RM1 cells were inoculated into mouse tibia, and tumor cells were separated that metastasized to lung tissue and again used tibial inoculation of RM1 cells; RM1‐LM3 cells were thus isolated from the tumor metastasized to lung tissue after three cycles in vivo. All cell lines tested negative for mycoplasma with the MycoFluor kit (Invitrogen). Each cell line was cultured in RPMI‐1640 medium with 10% fetal bovine serum and 1x penicillin/streptomycin at 37 °C in 5% CO_2_ in air. Cells showing stable knockdown of SMC4 were created with the CRISPR/Cas9 system per the previous publication.^[^ ^38^ ^]^ Briefly, the sgRNA (ATTATTGGACCAAATGGCAG) was phosphorylated and introduced into the pX459 plasmid containing mCherry (a gift from Nanjing Keris Biotechnology Company) to generate the Cas9‐sgRNA targeting plasmids for SMC4. Approximately 5 µg of the SMC4‐Cas9‐sgRNA plasmids were transfected into 1 × 10^6^ RM1‐LM3 cells using a cell line nucleofector kit (Lonza). Approximately 2 days after transfection, cells were sorted by flow cytometry (BD FACS Aria 5) and seeded into 96‐well plates at one cell per well density. Cell colonies were harvested and detected by PCR, and nucleotides were sequenced.

### siRNA Transfection

The specific siRNA for SMC4 (human) and control siRNA were designed and synthesized by GenePharma, and the oligo sequence for siRNA is shown in Table S2 (Supporting Information). Cells were transfected with oligonucleotides using Lipofectamine IMAX (Invitrogen Life Technologies), and after 48 h of transfection, the cells were digested and confirmed by RT‐qPCR or western blotting analysis.

### Cell‐Proliferation, Colony‐Formation, and Wound‐Healing Assays

For cell proliferation, cells were plated at a density of 1000 cells per well in a complete medium in 96‐well plates. Cell viability was assessed using the MTT assay at 24, 48, and 72 h; and measured absorbance at 450 nm with a spectrophotometric plate reader (BioTek). For colony formation, cells were plated at a density of 500 cells per well in a complete medium in six‐well plates. Ten to fourteen days later, the colonies were fixed with 4% paraformaldehyde and stained with 0.1% crystal violet, and the number of colonies was counted under an inverted microscope (Nikon Corporation). Regarding wound healing, cells were seeded into six‐well plates at a density of 5 × 10^5^ and allowed them to grow until >95% confluence. The monolayer was gently and slowly scraped with a new 200‐µl pipette tip across the center of the well, gently washed the wells twice with PBS to remove detached or dead cells, and replenished the wells with fresh serum‐free RPMI‐1640 medium. After the treatment, the same field in each well was imaged with a microscope (Nikon Corporation) at four time points (0, 10, 18, and 28 h). Images were processed and analyzed using ImageJ software, and the scratch areas of each observation point were measured at different times to calculate the cell migration rate.

### Cell‐Migration and Invasion Assays

With respect to cell migration, 6 × 10^4^ cells were resuspended in the RPMI‐1640 medium, seeded them into the top chamber of Transwell inserts (Corning Star), and added 15% FBS medium to the bottom chamber. Cells were incubated in chambers for 24 h, fixed with 4% paraformaldehyde for 20 min, and stained with 0.1% crystal violet. Non‐invading cells were removed using a cotton swab. Wells were imaged with five randomly selected fields counted under light microscopy and assayed in triplicate.

To determine cell invasion, 8 × 10^4^ cells were resuspended in RPMI‐1640 medium and seeded into the top chamber of a Matrigel‐coated Transwell inserts (Corning Star), and 15% FBS medium was added to the bottom chamber. Cells were incubated in chambers for 24 h, fixed in 4% paraformaldehyde for 20 min, and stained with 0.1% crystal violet. Non‐invading cells were removed using a cotton swab. Wells were imaged with five randomly selected fields counted under a microscope in triplicate assessments.

### Cell‐Cycle Assay

The percentages of cells within different phases of the cell cycle were evaluated by determining DNA content followed by propidium iodide (PI) staining (BD Biosciences). Briefly, cells were digested and washed with phosphate‐buffered saline (PBS), centrifuged at 1000 rpm for 10 min, fixed in 70–80% ethanol, and incubated at −20 °C for a minimum of 2 h. The cells were washed twice, re‐centrifuged the fixed cells, resuspended the cells in 0.5 mL of PI/RNase staining buffer, and incubated them for 15 min at room temperature. At least 20 000 cells were analyzed with a FACSCalibur instrument (BD Biosciences) equipped with Cell Quest 3.3 software.

### RNA Extraction and Quantitative Real‐Time PCR (qRT‐PCR)

Total RNA was extracted using TRIzol (Invitrogen), the concentration and purity were determined using a NanoDrop spectrophotometer (Thermo Scientific), and reverse transcription was executed with Takara Reverse Transcription Reagents. Quantitative real‐time PCR was performed in triplicate using a real‐time PCR System (Applied Biosystems) and SYBR Green PCR Master mix (Takara). Each experiment was repeated three times, and the sequences of the primers used are presented in Table S2 (Supporting Information).

### Western Immunoblotting Analysis

Western blotting analyses were performed using standard protocols. Briefly, total proteins were isolated from whole cells using RIPA with protease inhibitors, and protein extracts were separated on 10% SDS‐PAGE gels and electrophoretically transferred to nitrocellulose membranes. The membranes were blocked with 5% BSA in TBST buffer, incubated with primary antibodies, and protein bands were visualized using enhanced chemiluminescence detection. The primary antibodies used in western blotting were generated against SMC4 (Abcam), GLUT1 (Cell Signaling Technology), HK2 (Cell Signaling Technology), CDK2 (Cell Signaling Technology), Cdc2 (Cell Signaling Technology), HIF1a (Cell Signaling Technology), Flag (Cell Signaling Technology), HA (Cell Signaling Technology), P27 (Cell Signaling Technology), t‐mTOR (Cell Signaling Technology), CyclinB1 (Cell Signaling Technology), p‐mTOR (Phospho ser2448, Cell Signaling Technology), p70S6K (Cell Signaling Technology), Rheb (Abcam) and actin (Cell Signaling Technology); and the secondary antibodies were anti‐rabbit‐HRP (Cell Signaling Technology) and anti‐mouse‐HRP (Cell Signaling Technology).

### Co‐Immunoprecipitation

Total proteins were isolated from whole cells using NP‐40 buffer (Beyotime, #P0013F). Cell lysates were incubated with 2 ug of SMC4 antibody (1:100) followed by 20 µl of protein A + G agarose and incubated the mixture at 4 °C for 2 h. The supernatant was removed, and the beads were washed three times with NP‐40 buffer. Finally, the proteins bound to the beads were denatured using a 2× loading buffer and boiled. Then, the samples were evaluated using SDS–PAGE and western blotting.

### Immunofluorescence Staining

Cells were fixed with 4% (v/v) paraformaldehyde for 15 min, followed by permeabilization with 0.1% (w/v) Triton X‐100 for 10 min. After being blocked with 10% normal goat serum in PBS for 1 h at room temperature, the cells were incubated with SMC4 (Abcam) and GLUT1 (Proteintech) primary antibodies at 4 °C overnight. The primary antibodies were removed, and the cells were washed 3 times and incubated with species‐specific secondary antibodies for 1 h at room temperature in the dark. The secondary antibodies were removed, and the cells were washed 3 times and mounted. Images were acquired using the Abberior STEDYCON (Abberior Instruments) fluorescence microscope with a motorized inverted microscope IX83 (Olympus UPlanXAPO 100x). All images were analyzed using the STEDYCON gallery software (Abberior Instruments).

### Animal Experiments

C57BL/6 mice (4 weeks old) placed in the experiment were obtained from the Shanghai Model Organisms Center, Inc., Shanghai, China. Cells (8 × 10^4^) were resuspended in 0.02 mL of mixed medium (DMEM: Matrigel = 1:1) and injected into mouse tibias. The mice were weighed every 2–3 days and measured fluorescence every 5–7 days. At about 3 weeks, the mice were euthanized, and lungs were surgically dissected. The Southern University of Science and Technology Animal Care and Use Committee approved all experimental animal procedures.

### Immunohistochemistry

Mice were sacrificed under CO_2_ inhalation, and the lungs were removed and fixed in 4% PFA for 24 h under deep anesthesia. The fixed lungs were embedded in paraffin and cut at 4 µm with a sliding microtome (Leica RM2255), and the sections were mounted on glass slides. Immunohistochemical staining was conducted as previously described using an enzyme‐labeled biotin/streptavidin system and a solvent‐resistant DAB Map Kit. Primary antibodies used in this study were anti‐SMC4 (Abcam) and anti‐Ki67 (Cell Signaling Technology).

### RNA Sequencing and Bioinformatics Analysis

Total RNA was extracted using a TRIzol‐based kit (Thermo), and sequenced nucleotides with an Illumina HiSeq PE150 instrument and Promega Relia‐Prep. Paired‐end reads were obtained, the quality score was determined with FASTQC, and data were mapped to the GRCm/mm10 mouse genome assembly with STAR Aligner (version 2.5.0a). Differentially expressed genes (DEGs) with a false‐discovery rate (FDR) of < 0.05 were considered to constitute statistical significance. Pathway‐enrichment analysis was conducted using Gene Set Enrichment Analysis (GSEA) against the Molecular Signature Database (MSigDB), complying with the pathway compendium.

### Metabolic Analysis

An ATP Rate Kit (Seahorse), Glycolysis Ratio Kit (Seahorse), and Mito‐Stress Assay Kit (Seahorse) were used in this experiment according to the manufacturer's instructions. In brief, 1.5 × 10^4^ cells were seeded in a XF96 Cell Culture Microplate (Seahorse) in 80 µl of complete medium and incubated overnight. The cell growth medium was aspirated and changed to an XF assay medium, incubating the cells in a 37 °C CO_2_‐free incubator for 45 min. Extracellular acidification rate (ECAR) and oxygen consumption rate (OCR) were measured using a Seahorse XF96 Analyzer followed by the addition of corresponding reagents as described in the manufacturer's instructions. For the ATP rate assay, oligomycin (1.5 µm) and rotenone/antimycin A (Rot/AA, 0.5 µm) were serially injected onto plates; for the glycolytic rate assay, 10 mm glucose, 1 µm oligomycin, and 50 mm 2‐deoxy‐glucose (2‐DG) were added sequentially; and for the Mito‐Stress assay, 1.5 µm oligomycin, 0.5 µm FCCP, and 0.5 µm Rot/AA were serially added. Each plotted value is the mean of at least five multiple wells and is normalized to baseline OCR and total protein levels. The results were analyzed using Wave software (Seahorse), and data are presented as means ± SD. Significance was calculated using two‐way ANOVA with Tukey's multiple‐comparison test.

### Statistical Analysis

GraphPad Prism 7.0 software was used for graphical figures and statistical analyses. All data are expressed as means ± SEM. Student's t‐test was used to assess significant differences in two‐group comparisons; one‐way ANOVA was used for more than two groups. Spearman correlation was used to analyze correlations between two indicators. *p* values < 0.5 were regarded as statistically significant.

### Ethics Approval and Consent to Participate

The animal study was approved by the Institutional Animal Care and Use Committee of the Southern University of Science and Technology (SUSTC‐JY2018049).

## Conflict of Interest

The authors declare no conflict of interest.

## Author Contributions

W.Z., S.Q., and X.L. contributed equally to this work. J.Z., Y.L., and W.Z. designed the study; W.Z., M.C., and H.B.T. performed the experiments and collected all data; S.Y.Q. and X.K.L. analyzed and processed the data; W.Z., T.Z.W., and X.H. participated in the writing and figure generation. J.Z., Y.L., and X.G. critically reviewed the manuscript. All authors have read and approved the manuscript.

## Supporting information



Supporting Information

## Data Availability

The data that support the findings of this study are available in the supplementary material of this article.
