# Supplementary material for: SMC4 Promotes Prostate Cancer Cell Proliferation and Metastasis via the Rheb/mTOR Pathway
Source: Adv Sci (Weinh). 2025 Apr 25;12(23):2500369. doi: 10.1002/advs.202500369 (PMC12199409; doi:10.1002/advs.202500369)
Supplement: Supplementary file 1 — Supporting Information [file ADVS-12-2500369-s001.docx]

Supplementary figures


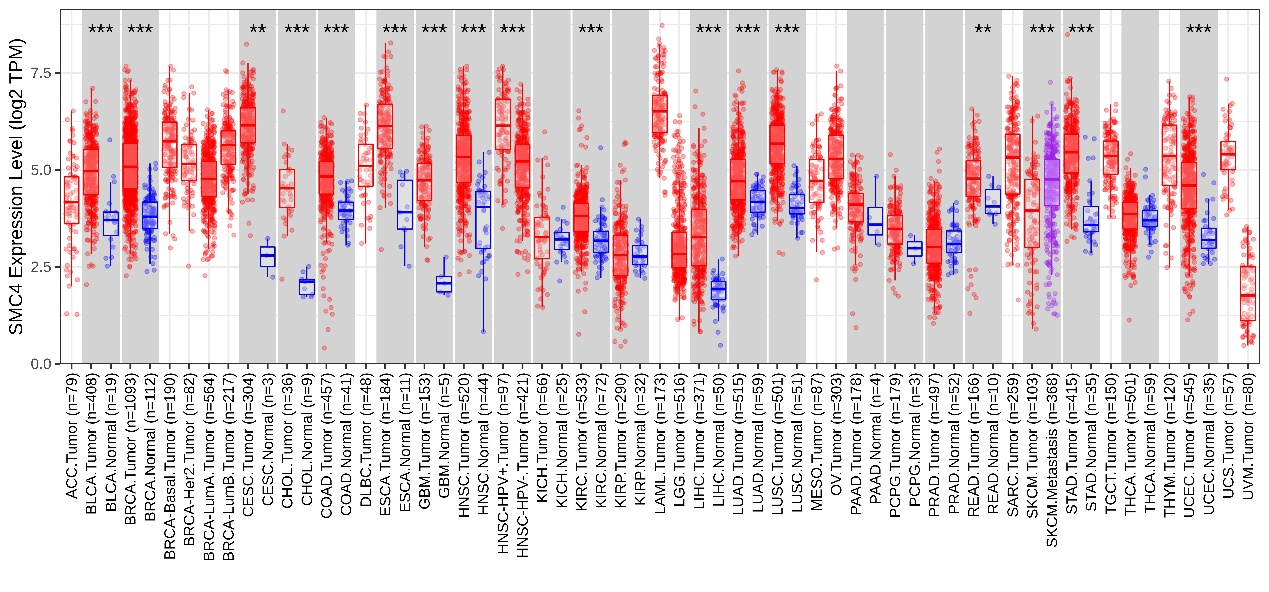


Fig. S1 Aberrant expression of SMC4 in tumors in the TCGA dataset


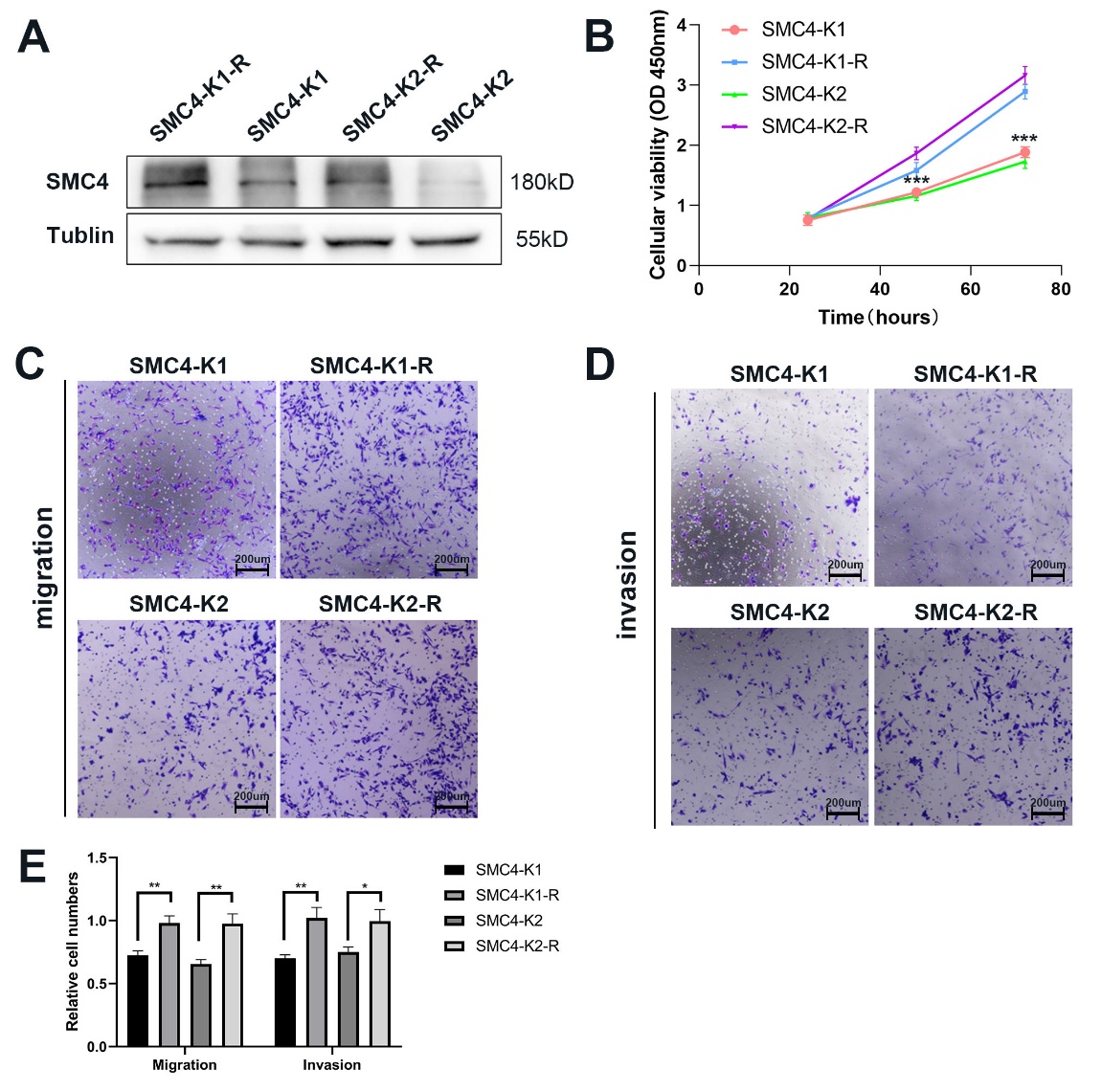


Figure S2 Overexpression of SMC4 promotes cell proliferation, migration, and invasion

A: Western blotting analysis of the protein expression of SMC4.

B: A CCK8 assay was executed to measure the proliferation of RM1-LM3 and SMC4 knockdown cells (data are shown as the mean ± SEM of triplicate wells and represent at least three replicate experiments).

C: Transwell assays were performed to determine cell migration.

D: Transwell assays were performed to determine cell invasion.

(SMC4-K1-R: SMC4-K1 cells overexpressing SMC4. SMC4-K2-R: SMC4-K1 cells overexpressing SMC4.)

E：Relative analysis of migration and invasion.

***P < 0.001 based on Student t test; error bars signify means ± SEM of three independent experiments.


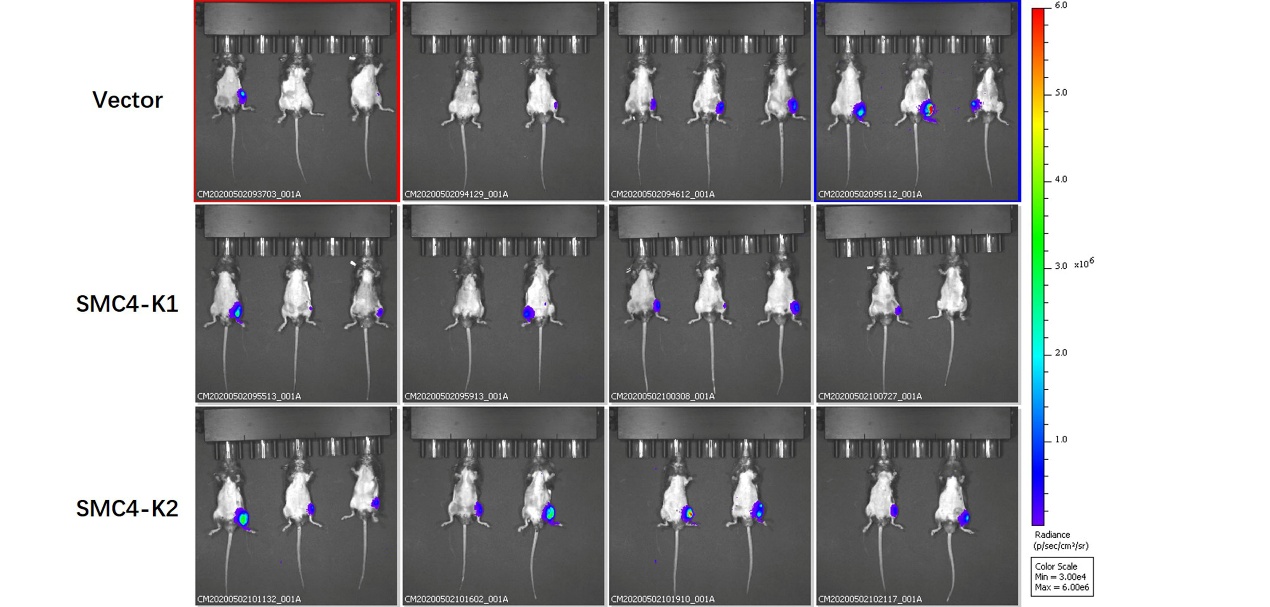


Fig. S3 Bioluminescence imaging (BLI) of mouse tumors was measured at day 7.


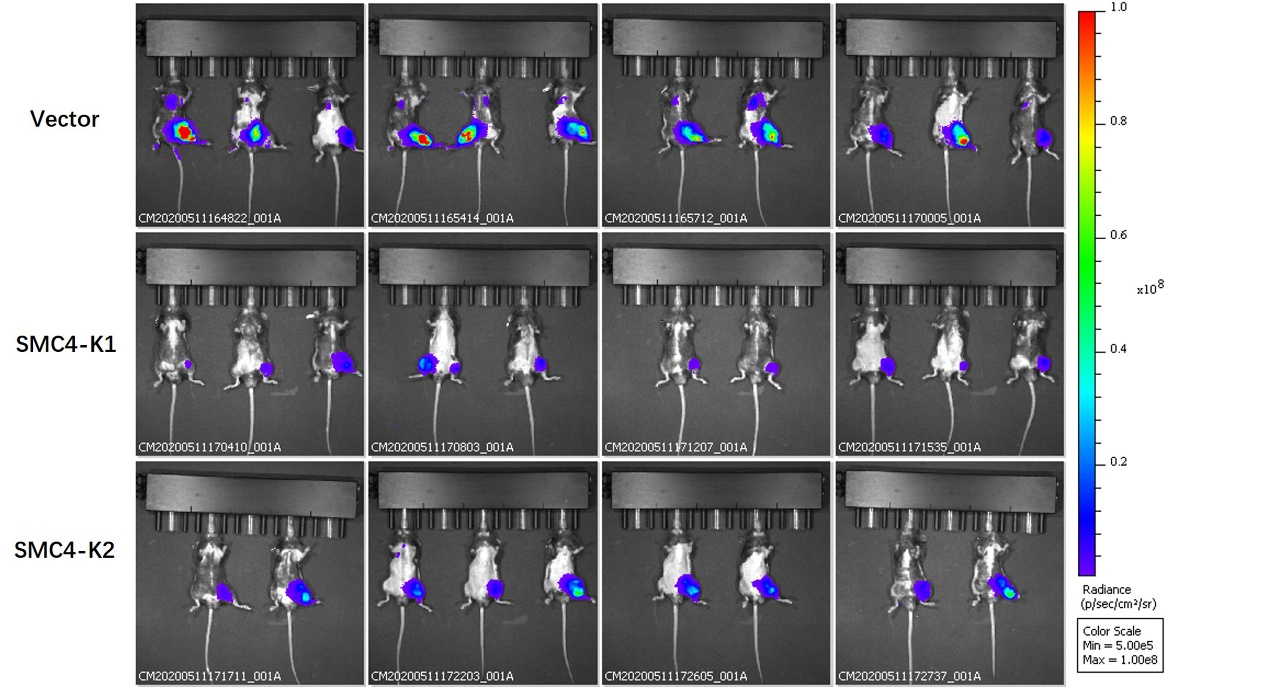


Fig. S4 Bioluminescence imaging (BLI) of mouse tumors was measured at day 14.


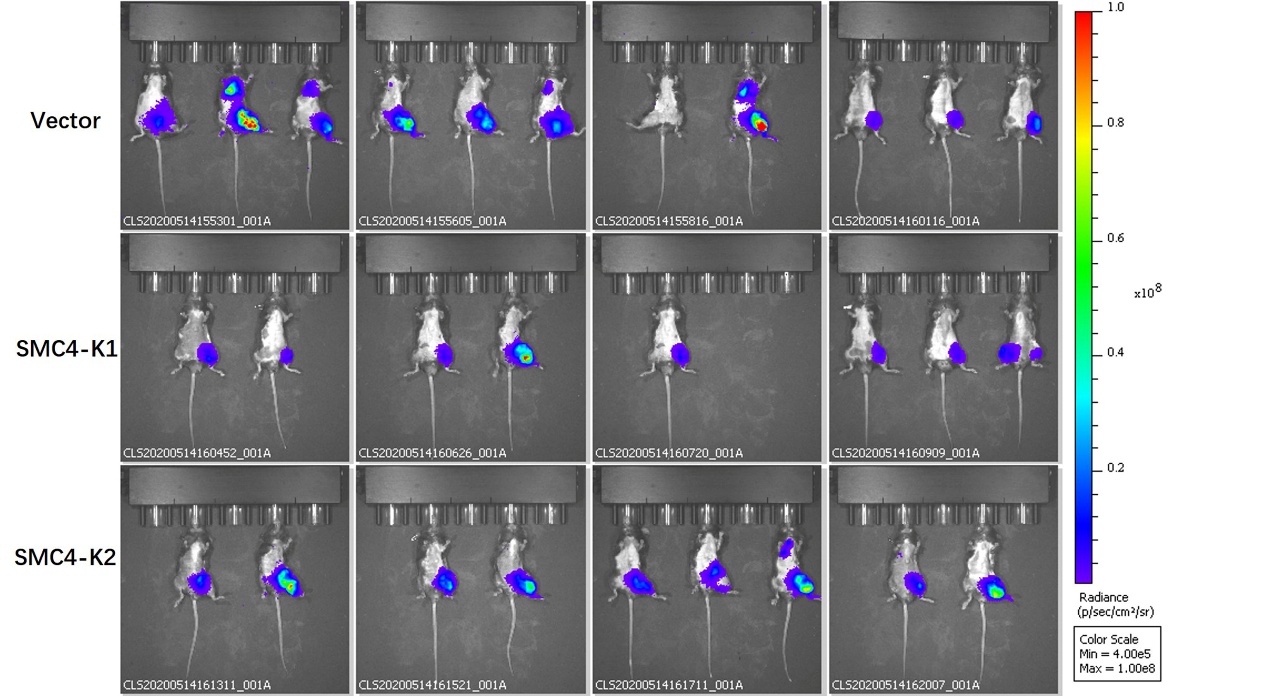


Fig. S5 Bioluminescence imaging (BLI) of mouse tumors was measured at day 19.


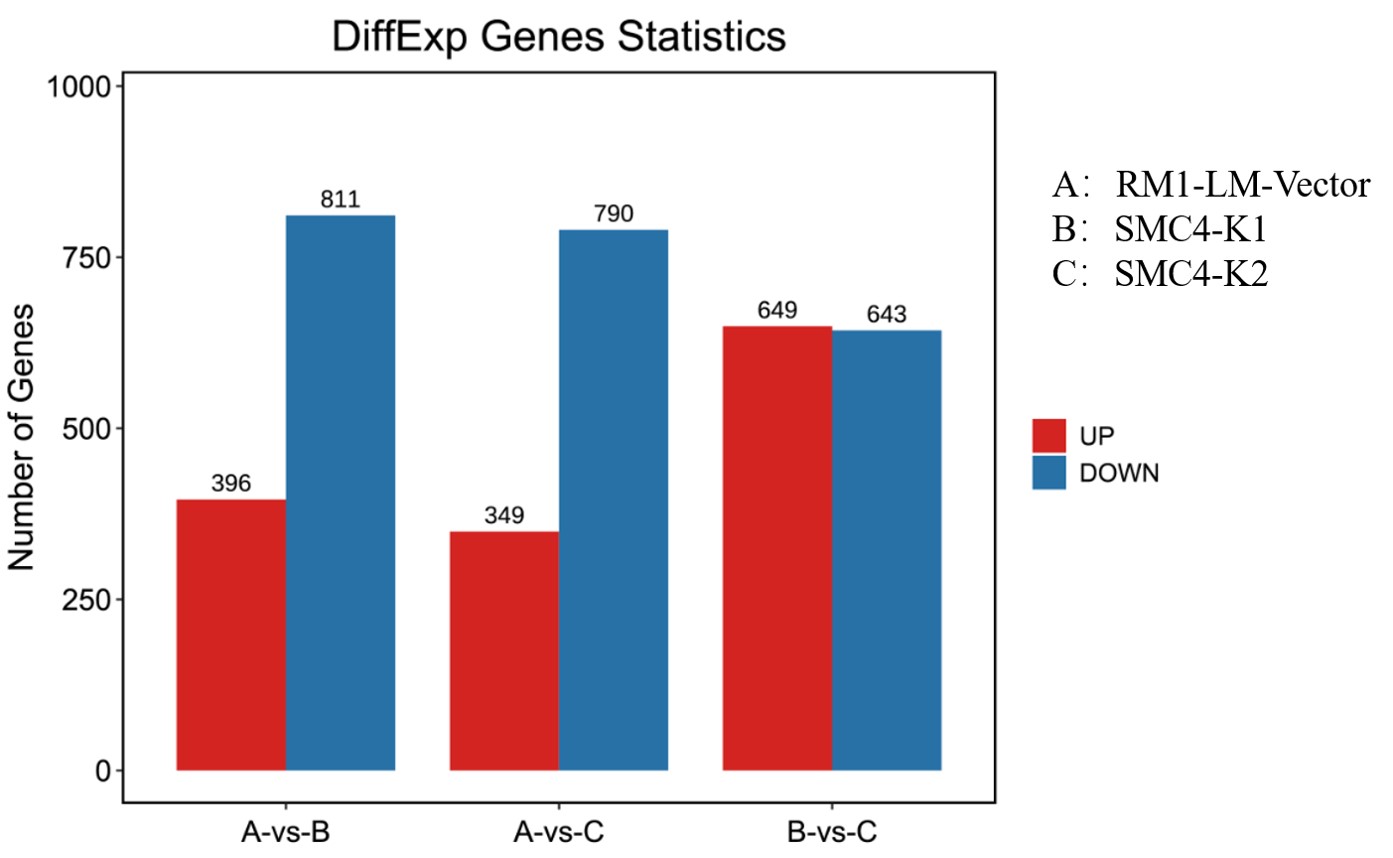


Figure S6 Differentially expressed gene statistics


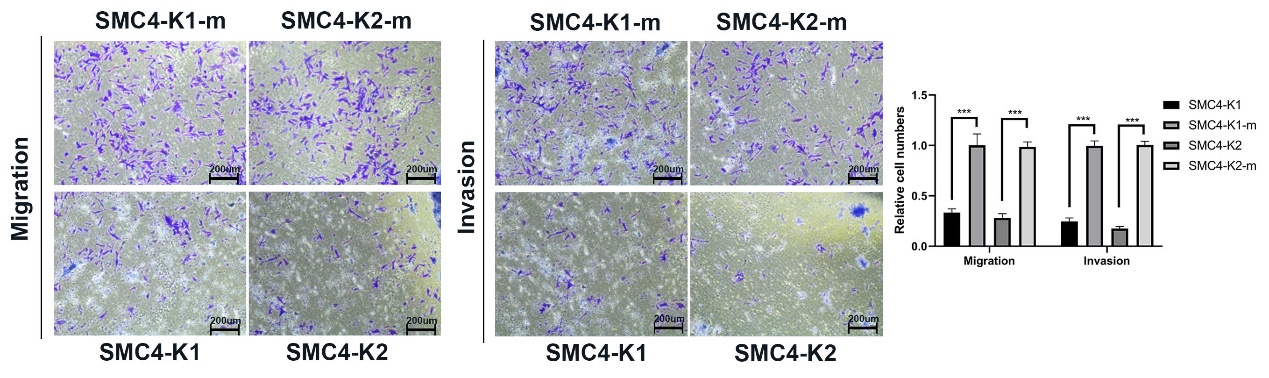


Figure S7 Cell migration and invasion capacities were increased after treatment with an mTOR activator. (SMC4-K1-m: SMC4-K1 cells treated with mTOR activator. SMC4-K2-m: SMC4-K2 cells treated with mTOR activator.)

Tables

Table S1 A total of 76 proteins were identified as interacting specifically with Flag-SMC4

| Accession | Description |
| --- | --- |
| O08852\|PKD1_MOUSE | Polycystin-1 OS=Mus musculus GN=Pkd1 PE=1 SV=2 |
| O35691\|PININ_MOUSE | Pinin OS=Mus musculus GN=Pnn PE=1 SV=4 |
| Q8CG48\|SMC2_MOUSE | Structural maintenance of chromosomes protein 2 OS=Mus musculus GN=Smc2 PE=1 SV=2 |
| P11983\|TCPA_MOUSE | T-complex protein 1 subunit alpha OS=Mus musculus GN=Tcp1 PE=1 SV=3 |
| Q8CCS6\|PABP2_MOUSE | Polyadenylate-binding protein 2 OS=Mus musculus GN=Pabpn1 PE=1 SV=3 |
| Q6PDK2\|KMT2D_MOUSE | Histone-lysine N-methyltransferase 2D OS=Mus musculus GN=Kmt2d PE=1 SV=2 |
| Q9R0H5\|K2C71_MOUSE | Keratin type II cytoskeletal 71 OS=Mus musculus GN=Krt71 PE=1 SV=1 |
| Q9QYX7\|PCLO_MOUSE | Protein piccolo OS=Mus musculus GN=Pclo PE=1 SV=4 |
| B2RRE7\|OTUD4_MOUSE | OTU domain-containing protein 4 OS=Mus musculus GN=Otud4 PE=1 SV=1 |
| P43274\|H14_MOUSE | Histone H1.4 OS=Mus musculus GN=Hist1h1e PE=1 SV=2 |
| Q8CG47\|SMC4_MOUSE | Structural maintenance of chromosomes protein 4 OS=Mus musculus GN=Smc4 PE=1 SV=1 |
| Q8BJ37\|TYDP1_MOUSE | Tyrosyl-DNA phosphodiesterase 1 OS=Mus musculus GN=Tdp1 PE=1 SV=2 |
| P11881\|ITPR1_MOUSE | Inositol 1 4 5-trisphosphate receptor type 1 OS=Mus musculus GN=Itpr1 PE=1 SV=2 |
| P14733\|LMNB1_MOUSE | Lamin-B1 OS=Mus musculus GN=Lmnb1 PE=1 SV=3 |
| P63276\|RS17_MOUSE | 40S ribosomal protein S17 OS=Mus musculus GN=Rps17 PE=1 SV=2 |
| Q07643\|CO9A2_MOUSE | Collagen alpha-2(IX) chain OS=Mus musculus GN=Col9a2 PE=2 SV=1 |
| Q08943\|SSRP1_MOUSE | FACT complex subunit SSRP1 OS=Mus musculus GN=Ssrp1 PE=1 SV=2 |
| Q9D6P8\|CALL3_MOUSE | Calmodulin-like protein 3 OS=Mus musculus GN=Calml3 PE=2 SV=1 |
| P60867\|RS20_MOUSE | 40S ribosomal protein S20 OS=Mus musculus GN=Rps20 PE=1 SV=1 |
| P0DP28\|CALM3_MOUSE | Calmodulin-3 OS=Mus musculus GN=Calm3 PE=1 SV=1 |
| P0DP27\|CALM2_MOUSE | Calmodulin-2 OS=Mus musculus GN=Calm2 PE=1 SV=1 |
| P0DP26\|CALM1_MOUSE | Calmodulin-1 OS=Mus musculus GN=Calm1 PE=1 SV=1 |
| Q8BX17\|GEMI5_MOUSE | Gem-associated protein 5 OS=Mus musculus GN=Gemin5 PE=1 SV=2 |
| Q3V0Q1\|DYH12_MOUSE | Dynein heavy chain 12 axonemal OS=Mus musculus GN=Dnah12 PE=1 SV=2 |
| Q9Z1X4\|ILF3_MOUSE | Interleukin enhancer-binding factor 3 OS=Mus musculus GN=Ilf3 PE=1 SV=2 |
| P23475\|XRCC6_MOUSE | X-ray repair cross-complementing protein 6 OS=Mus musculus GN=Xrcc6 PE=1 SV=5 |
| Q9QYJ0\|DNJA2_MOUSE | DnaJ homolog subfamily A member 2 OS=Mus musculus GN=Dnaja2 PE=1 SV=1 |
| Q99NH2\|PARD3_MOUSE | Partitioning defective 3 homolog OS=Mus musculus GN=Pard3 PE=1 SV=2 |
| P70193\|LRIG1_MOUSE | Leucine-rich repeats and immunoglobulin-like domains protein 1 OS=Mus musculus GN=Lrig1 PE=1 SV=2 |
| Q62095\|DDX3Y_MOUSE | ATP-dependent RNA helicase DDX3Y OS=Mus musculus GN=Ddx3y PE=1 SV=2 |
| Q7TQH0\|ATX2L_MOUSE | Ataxin-2-like protein OS=Mus musculus GN=Atxn2l PE=1 SV=1 |
| P28740\|KIF2A_MOUSE | Kinesin-like protein KIF2A OS=Mus musculus GN=Kif2a PE=1 SV=2 |
| E9Q5G3\|KIF23_MOUSE | Kinesin-like protein KIF23 OS=Mus musculus GN=Kif23 PE=1 SV=1 |
| Q8C8U0\|LIPB1_MOUSE | Liprin-beta-1 OS=Mus musculus GN=Ppfibp1 PE=1 SV=3 |
| Q8VDJ3\|VIGLN_MOUSE | Vigilin OS=Mus musculus GN=Hdlbp PE=1 SV=1 |
| Q9CQS8\|SC61B_MOUSE | Protein transport protein Sec61 subunit beta OS=Mus musculus GN=Sec61b PE=1 SV=3 |
| Q60865\|CAPR1_MOUSE | Caprin-1 OS=Mus musculus GN=Caprin1 PE=1 SV=2 |
| Q99JP6\|HOME3_MOUSE | Homer protein homolog 3 OS=Mus musculus GN=Homer3 PE=1 SV=2 |
| Q9QYC0\|ADDA_MOUSE | Alpha-adducin OS=Mus musculus GN=Add1 PE=1 SV=2 |
| Q9Z108\|STAU1_MOUSE | Double-stranded RNA-binding protein Staufen homolog 1 OS=Mus musculus GN=Stau1 PE=1 SV=1 |
| Q3UGY8\|BIG3_MOUSE | Brefeldin A-inhibited guanine nucleotide-exchange protein 3 OS=Mus musculus GN=Arfgef3 PE=1 SV=1 |
| Q921F2\|TADBP_MOUSE | TAR DNA-binding protein 43 OS=Mus musculus GN=Tardbp PE=1 SV=1 |
| P17809\|GTR1_MOUSE | Solute carrier family 2 facilitated glucose transporter member 1 OS=Mus musculus GN=Slc2a1 PE=1 SV=4 |
| Q8BMK4\|CKAP4_MOUSE | Cytoskeleton-associated protein 4 OS=Mus musculus GN=Ckap4 PE=1 SV=2 |
| Q9WVR4\|FXR2_MOUSE | Fragile X mental retardation syndrome-related protein 2 OS=Mus musculus GN=Fxr2 PE=1 SV=1 |
| Q9D6Z1\|NOP56_MOUSE | Nucleolar protein 56 OS=Mus musculus GN=Nop56 PE=1 SV=2 |
| Q62189\|SNRPA_MOUSE | U1 small nuclear ribonucleoprotein A OS=Mus musculus GN=Snrpa PE=1 SV=3 |
| P27773\|PDIA3_MOUSE | Protein disulfide-isomerase A3 OS=Mus musculus GN=Pdia3 PE=1 SV=2 |
| P05784\|K1C18_MOUSE | Keratin type I cytoskeletal 18 OS=Mus musculus GN=Krt18 PE=1 SV=5 |
| P97479\|MYO7A_MOUSE | Unconventional myosin-VIIa OS=Mus musculus GN=Myo7a PE=1 SV=2 |
| Q9QYB8\|ADDB_MOUSE | Beta-adducin OS=Mus musculus GN=Add2 PE=1 SV=4 |
| P21981\|TGM2_MOUSE | Protein-glutamine gamma-glutamyltransferase 2 OS=Mus musculus GN=Tgm2 PE=1 SV=4 |
| Q9DB34\|CHM2A_MOUSE | Charged multivesicular body protein 2a OS=Mus musculus GN=Chmp2a PE=1 SV=1 |
| O88554\|PARP2_MOUSE | Poly [ADP-ribose] polymerase 2 OS=Mus musculus GN=Parp2 PE=1 SV=3 |
| Q8K2Z4\|CND1_MOUSE | Condensin complex subunit 1 OS=Mus musculus GN=Ncapd2 PE=1 SV=2 |
| Q3UMG5\|LRCH2_MOUSE | Leucine-rich repeat and calponin homology domain-containing protein 2 OS=Mus musculus GN=Lrch2 PE=2 SV=2 |
| Q6DFW4\|NOP58_MOUSE | Nucleolar protein 58 OS=Mus musculus GN=Nop58 PE=1 SV=1 |
| Q569Z6\|TR150_MOUSE | Thyroid hormone receptor-associated protein 3 OS=Mus musculus GN=Thrap3 PE=1 SV=1 |
| Q61584\|FXR1_MOUSE | Fragile X mental retardation syndrome-related protein 1 OS=Mus musculus GN=Fxr1 PE=1 SV=2 |
| P26039\|TLN1_MOUSE | Talin-1 OS=Mus musculus GN=Tln1 PE=1 SV=2 |
| Q925I1\|ATAD3_MOUSE | ATPase family AAA domain-containing protein 3 OS=Mus musculus GN=Atad3 PE=1 SV=1 |
| Q8R050\|ERF3A_MOUSE | Eukaryotic peptide chain release factor GTP-binding subunit ERF3A OS=Mus musculus GN=Gspt1 PE=1 SV=2 |
| Q149F3\|ERF3B_MOUSE | Eukaryotic peptide chain release factor GTP-binding subunit ERF3B OS=Mus musculus GN=Gspt2 PE=1 SV=1 |
| Q99KD5\|UN45A_MOUSE | Protein unc-45 homolog A OS=Mus musculus GN=Unc45a PE=1 SV=2 |
| Q3TIV5\|ZC3HF_MOUSE | Zinc finger CCCH domain-containing protein 15 OS=Mus musculus GN=Zc3h15 PE=1 SV=2 |
| Q9QWT9\|KIFC1_MOUSE | Kinesin-like protein KIFC1 OS=Mus musculus GN=Kifc1 PE=1 SV=2 |
| Q8VDM6\|HNRL1_MOUSE | Heterogeneous nuclear ribonucleoprotein U-like protein 1 OS=Mus musculus GN=Hnrnpul1 PE=1 SV=1 |
| P19096\|FAS_MOUSE | Fatty acid synthase OS=Mus musculus GN=Fasn PE=1 SV=2 |
| Q1HFZ0\|NSUN2_MOUSE | tRNA (cytosine(34)-C(5))-methyltransferase OS=Mus musculus GN=Nsun2 PE=1 SV=2 |
| P32067\|LA_MOUSE | Lupus La protein homolog OS=Mus musculus GN=Ssb PE=1 SV=1 |

Table S2 qPCR primers used in this study

| Species | Name | | Sequence (5'-3') |
| --- | --- | --- | --- |
| *Mus musculus* | Actin | Forward | CGTTGACATCCGTAAAGACC |
|  |  | Reverse | AACAGTCCGCCTAGAAGCAC |
|  | SMC4 | Forward | AATAGTATCCCTCCACCC |
|  |  | Reverse | GCTCGATAGCCAAACA |
| *Homo sapiens* | Actin | Forward | CATGTACGTTGCTATCCAGGC |
|  |  | Reverse | CTCCTTAATGTCACGCACGAT |
|  | SMC4 | Forward | TCCTATGCTGGGGAGAAA |
|  |  | Reverse | CAATAACATTGGATTTGC |
